# Antibody–Drug Conjugate αEGFR-E-P125A Reduces Triple-negative Breast Cancer Vasculogenic Mimicry, Motility, and Metastasis through Inhibition of EGFR, Integrin, and FAK/STAT3 Signaling

**DOI:** 10.1158/2767-9764.CRC-23-0278

**Published:** 2024-03-11

**Authors:** Ankita P. Sankar, Hyun-Mi Cho, Seung-Uon Shin, Tal Sneh, Sundaram Ramakrishnan, Christian Elledge, Yu Zhang, Rathin Das, Hava Gil-Henn, Joseph D. Rosenblatt

**Affiliations:** 1 Department of Medicine, Division of Hematology, University of Miami Miller School of Medicine, Miami, Florida.; 2 Sylvester Comprehensive Cancer Center, Miami, Florida.; 3 The Azrieli Faculty of Medicine, Bar-Ilan University, Safed, Israel.; 4 Dewitt Daughtry Department of Surgery, University of Miami Miller School of Medicine, Miami, Florida.; 5 Synergys Biotherapeutics, Inc., Alamo, California.

## Abstract

Primary tumor growth and metastasis in triple-negative breast cancer (TNBC) require supporting vasculature, which develop through a combination of endothelial angiogenesis and vasculogenic mimicry (VM), a process associated with aggressive metastatic behavior in which vascular-like structures are lined by tumor cells. We developed αEGFR-E-P125A, an antibody-endostatin fusion protein that delivers a dimeric, mutant endostatin (E-P125A) payload that inhibits TNBC angiogenesis and VM *in vitro* and *in vivo*. To characterize the mechanisms associated with induction and inhibition of VM, RNA sequencing (RNA-seq) of MDA-MB-231-4175 TNBC cells grown in a monolayer (two-dimensional) was compared with cells plated on Matrigel undergoing VM [three-dimensional (3D)]. We then compared RNA-seq between TNBC cells in 3D and cells in 3D with VM inhibited by αEGFR-E-P125A (EGFR-E-P125A). Gene set enrichment analysis demonstrated that VM induction activated the IL6-JAK-STAT3 and angiogenesis pathways, which were downregulated by αEGFR-E-P125A treatment.

Correlative analysis of the phosphoproteome demonstrated decreased EGFR phosphorylation at Y1069, along with decreased phosphorylation of focal adhesion kinase Y397 and STAT3 Y705 sites downstream of α5β1 integrin. Suppression of phosphorylation events downstream of EGFR and α5β1 integrin demonstrated that αEGFR-E-P125A interferes with ligand-receptor activation, inhibits VM, and overcomes oncogenic signaling associated with EGFR and α5β1 integrin cross-talk.

*In vivo*, αEGFR-E-P125A treatment decreased primary tumor growth and VM, reduced lung metastasis, and confirmed the inhibition of signaling events observed *in vitro*. Simultaneous inhibition of EGFR and α5β1 integrin signaling by αEGFR-E-P125A is a promising strategy for the inhibition of VM, tumor growth, motility, and metastasis in TNBC and other EGFR-overexpressing tumors.

**Significance:**

αEGFR-E-P125A reduces VM, angiogenesis, tumor growth, and metastasis by inhibiting EGFR and α5β1 integrin signaling, and is a promising therapeutic agent for TNBC treatment, used alone or in combination with chemotherapy.

## Introduction

Triple-negative breast cancer (TNBC) is a highly aggressive and metastatic subtype of breast cancer, characterized by the absence of estrogen and progesterone receptors, and low levels of HER2 expression (1). Upregulation of EGFR expression and signaling is observed in 50%–60% of TNBC, *EGFR* amplification, or high copy number is reported in up to 30% of cases, and *EGFR* mutations in 11% of cases (2). Studies have correlated high *EGFR* copy number to EGFR protein overexpression, which is associated with poor clinical outcome in TNBC (3). Despite the prevalence of EGFR overexpression and/or high *EGFR* copy number, anti-EGFR therapies, such as cetuximab have limited therapeutic efficacy, emphasizing a need for more effective targeted therapies for the treatment of TNBC (4, 5).

Primary tumor growth and the formation of metastases in TNBC require the development of a supporting tumor vasculature that can occur through angiogenesis, the formation of new blood vessels from endothelial cells, or through the unique process of vasculogenic mimicry (VM), in which tubular channels lined by tumor cells rather than endothelial cells are formed (6). VM is commonly observed in TNBC and is associated with an increased propensity to metastasize (7–9). VM-positive tumors express mesenchymal markers and demonstrate increased expression and phosphorylation of transcription factors such as STAT3 and VM markers, including N-cadherin, VE-cadherin, Tie-1, matrix metalloproteases (MMP) 2 and 9, and focal adhesion kinase (FAK; ref. 10).

To create a more potent antiangiogenic therapy and target VM in TNBC, we previously developed an anti-EGFR antibody-endostatin fusion protein (αEGFR-E-P125A), constructed by fusing a mutated form of endostatin with enhanced endothelial binding and antitumor activity, HuEndoP125A (E-P125A), and an anti-EGFR antibody (11–14). Anti-EGFR antibodies, such as cetuximab, have proven to be effective in a variety of cancer types, including colorectal cancer, lung, and head and neck squamous cell carcinoma (15–17). The antiangiogenic agent endostatin showed antitumor activity in preclinical models (14) and clinically in breast cancer, sarcoma, and non–small cell lung cancers when used in combination with radiotherapy or chemotherapy (18, 19). However, neither cetuximab nor endostatin have demonstrated significant efficacy in patients with TNBC (4, 20, 21).

The efficacy of monotherapy is limited by compensatory signaling between growth factor receptors such as EGFR and integrin signaling. Cetuximab can prevent both dimerization and phosphorylation of EGFR (22). The use of cetuximab monotherapy to inhibit EGFR signaling leads to compensatory activation of α5β1 integrin, which may in turn promote tumor cell migration and metastasis (23–25). Endostatin inhibits the activation of α5β1 integrin–driven signaling (26). However, inhibition of signaling via α5β1 integrin alone may activate compensatory signaling via EGFR (27). Integrins, such as α5β1, can either directly or indirectly associate with growth factor receptors such as EGFR, to promote intracellular signaling leading to tumor growth and invasion. EGF-dependent regulation of the proto-oncogene Src in turn can activate α5β1 integrin (28–30). Disruption of the interplay between α5β1 integrin and EGFR signaling may be an effective means of reducing tumor cell motility and metastasis.

αEGFR-E-P125A delivers a dimeric endostatin payload and markedly inhibits tumor angiogenesis, TNBC VM, and tumor cell migration and metastasis *in vitro* and *in vivo* (12). We studied the mechanism of αEGFR-E-P125A action in TNBC and related the signaling changes induced by αEGFR-E-P125A treatment to the process of VM inhibition in MDA-MB-231-4175 TNBC *in vitro* and *in vivo*. We demonstrate that αEGFR-E-P125A simultaneously inhibits both EGFR and α5β1 integrin, with the anti-EGFR moiety inhibiting EGF-induced EGFR phosphorylation, and the E-P125A domain inhibiting fibronectin-induced FAK phosphorylation regulated by α5β1 integrin. Our findings demonstrated that simultaneous inhibition of EGFR and α5β1 integrin activation and signaling led to an overall decrease in VM and tumor cell motility *in vitro* and tumor growth and metastasis *in vivo*. These findings suggest that αEGFR-E-P125A is a promising therapeutic agent for TNBC treatment.

## Materials and Methods

### Cell Lines

Female MDA-MB-231-4175 human lung tropic TNBC cells (RRID: CVCL_5998) were gifted by J. Massagué, Memorial Sloan Kettering Cancer Center (31). Authentication of cells was performed by suppliers. TNBC cell lines were tested regularly for *Mycoplasma* using MycoAlert Mycoplasma detection kit (Lonza, catalog no. LT07-218) and cultured in RPMI1640 supplemented with 10% FBS and 100 U/mL penicillin–streptomycin (Gibco-BRL). Cell lines were grown to a maximum of 20 passages.

### Phospho-antibody Array

A total of 5.0 × 10^5^ MDA-MB-231-4175 cells were plated on Matrigel in a 12-well plate and treated with either a media control or αEGFR-E-P125A for 16 hours. Cells were lysed with RIPA buffer and protein samples were prepared. Protein lysates were biotinylated and incubated with the Phospho-Explorer antibody array (Full Moon Biosystems, catalog no. PEX100), and antibody expression was detected using dye-labeled Cy3 streptavidin (Sigma-Aldrich, catalog no. S6402). The slides were submitted to Full Moon Biosystems for reading and quantification.

### Tube Formation Assay

VM tube formation using TNBC tumor cells was performed as described previously (12). MDA-MB-231-4175 cells were resuspended in RPMI (Lonza) and plated on Matrigel-coated wells containing αEGFR-E-P125A or the control proteins as indicated. Following incubation for 16 hours at 37°C, tube formation was examined, and images were taken from the center of each well at 5x and 10x magnification using the phase contrast setting of the *Leica* DMIL microscope.

### Western Blot Analysis

A total of 5.0 × 10^5^ MDA-MB-231-4175 TNBC cells were plated on Matrigel in a 12-well plate. Following tube formation, the supernatant was aspirated and the wells were washed with 1 mL cold PBS (Gibco, catalog no. 10010023). PBS was removed and 2 mL of Cell Recovery solution was added to each well (Corning, catalog no. 354270). The mixture was then agitated for 2 hours at 4°C on a shaker. Following cell recovery, the cells and cell recovery solution were removed from the wells and transferred to a 15 mL conical tube on ice. The cells were washed with cold PBS and centrifuged at 1,200 rpm for 5 minutes. The supernatant was aspirated and the cells were washed and transferred to a microcentrifuge tube for centrifugation. The supernatant was aspirated, and the cell pellet was resuspended in 100 mL RIPA buffer for 30 minutes, followed by vortexing and centrifugation for 10 minutes at 15,000 rpm at 4°C. The supernatant was collected for the determination of the protein concentration.

### Immunofluorescence Staining

Coverslips (VWR, catalog no. 48366067) were sterilized and placed in 6-well plates. Next, 150 mL Matrigel (Corning, catalog no. 354234) was added to the coverslips and incubated for 1 hour. One million MDA-MB-231-4175 cells were added to each well and incubated overnight for 16 hours at 37°C. The following day, the medium was aspirated from each well and the wells were washed 3x with PBS. For both cells and frozen xenograft tumor tissues, samples were fixed with 2 mL of 4% paraformaldehyde for 30 minutes, followed by permeabilization with Tween 20 for 15 minutes. The wells were washed once and blocked with 2 mL of 5% BSA for 1 hour at room temperature. Primary antibodies were added and the cells were incubated overnight at 4°C. The following day, the secondary antibody was added and incubated for 1–2 hours at room temperature in the dark. Wells were washed and coverslips were mounted onto slides using Fluoro-gel mounting medium containing DAPI (Electron Microscopy Sciences, catalog no. 17985-50). Slides were imaged at 10x and 20x magnification using a *Leica* DMIL LED fluorescent microscope.

### Immunoprecipitation

A total of 1 × 10^6^ MDA-MB-231-4175 cells were plated in 10 cm dishes in 10 mL RPMI media, and selected dishes were treated with the indicated equimolar doses of cetuximab, E-P125A, or αEGFR-E-P125A for 16 hours. The following day, the culture medium was removed, and the cells were washed once with PBS. Immunoprecipitation (IP) reagents were obtained from Pierce Classic IP Kit (Thermo Fisher Scientific, catalog no. 26146). IP Lysis/Wash Buffer was added to the cells on ice and incubated for 5 minutes with periodic mixing. The lysate was centrifuged and the cell debris was pelleted. The supernatant was collected for protein concentration determination and immunoprecipitated using 1 mg of cell lysate and α5 integrin antibody (Cell Signaling Technology, catalog no. 4705S) according to the manufacturer's instructions. The resulting IP eluate was used for Western blot analysis.

### Scratch Wound Migration Assay

MDA-MB-231-4175 cells were plated in a 96-well ImageLock Microplate (Essen BioScience, catalog no. 4379) at a density of 3.5 × 10^4^ cells/well in RPMI medium. Twenty-four hours after seeding, the WoundMaker (Essen BioScience, catalog no. 4563) was used to create reproducible scratch wounds in each well. The medium was aspirated after wounding, and the cells were washed and treated with αEGFR-E-P125A or equimolar amounts of the control treatments. The assay plate was incubated in an Incucyte ZOOM incubator (Essen BioScience), and wound width was monitored by imaging at the 10x objective every 4 hours. Wound width was characterized by the average distance between the top and bottom edges of the migrating cell population within the scratch wound (microns) and was computed using Incucyte software.

### siRNA Knockdown

For siRNA-mediated knockdown, control non-silencing siRNA (Sigma-Aldrich, catalog no. SIC001), EGFR siRNA (Thermo Fisher Scientific, catalog no. AM51331), ITGA5 siRNA (Sigma-Aldrich, catalog no. SASI_Hs01_00058581), and FAK siRNA (SMARTpool, L-003164-00) were used. A total of 2.0 × 10^5^ MDA-MB-231-4175 TNBC cells were plated in a 6-well plate 24 hours before transfection. On the day of transfection, cells were approximately 50% confluent. siRNA transfections were performed by combining 10 mL Xtreme-gene transfection reagent (Sigma-Aldrich, SITRAN-RO) diluted in 100 mL Opti-MEM serum-free media (Gibco, 31-985-070), with 10–20 nmol/L siRNA diluted in 100 mL Opti-MEM and incubated for 20 minutes at 15°C–25°C. The complex was added to 2 mL of medium and the mixture was then added to the plated cells for 24–48 hours before each experiment, depending on the specific gene of interest. Gene knockdown was validated using Western blotting.

### RNA Extraction

MDA-MB-231-4175 cells were plated in a two-dimensional (2D) monolayer, three-dimensional (3D) on Matrigel, or in 3D and treated with αEGFR-E-P125A for 16 hours in a 6-well cell culture dish. After 16 hours of incubation, the medium was aspirated, and the cells were washed twice with PBS. The cells were resuspended using TRIzol and homogenized by pipetting and vortexing. The samples were incubated at room temperature, and 200 mL of chloroform was added, followed by centrifugation at 4°C. Four biological replicates per treatment condition were independently treated on 4 consecutive days and frozen after 16 hours, and RNA from all replicates was isolated on the final day. Similarly, samples from tumor tissues were disrupted and homogenized using a TissueLyser in an appropriate volume of buffer RLT, the lysate was centrifuged, and the supernatant was collected. Ethanol (70%) was added to the supernatant of either the cells or tumor tissues and the samples were treated with DNase at room temperature for 15 minutes. RNA was isolated from all samples using the QIAGEN RNAeasy kit according to the manufacturer's instructions (QIAGEN, catalog no. 74004). The RNA concentration was quantified using Nanodrop and QUBIT, and the samples were then subjected to bulk RNA sequencing (RNA-seq) or NanoString nCounter analysis.

### RNA-seq Analysis

Principal component and differential gene expression analyses were performed at the Weizmann Institute of Science. Pairwise RNA-seq comparison during VM induction was performed by analyzing 2D versus 3D samples of MDA-MB-231-4175 cells. A separate comparison was performed by comparing the 3D αEGFR-E-P125A–treated samples with the 3D control RNA samples. Differentially expressed genes (DEG) were identified using a *P*-adjusted value ≤ 0.05, log_2_Fc ≥ 0.6, and max_count = 30. The entire gene set was input into the gene set enrichment analysis (GSEA) software using the Hallmarks gene set for further pathway analysis. Using RStudio, a combination of Pathview and SBGNview packages were used to investigate changes in the identified pathways of interest. Genes that were upregulated in the interrogated pathways are depicted in red and genes that were downregulated are depicted in green.

### Image J Analysis

The Angiogenesis Analyzer plug-in was used to quantify the number of meshes in tube formation assays. Immunofluorescence staining was quantified by analyzing the percent fluorescent area of each field within the stain, and IHC was analyzed using the H DAB staining setting within the Color Deconvolution function in FIJI. The density of Western blot bands was quantified using gel analysis feature with Image J. JaCoP colocalization plug-in was used to assess the colocalization of selected proteins.

### Ibidi Cell Motility Tracker

Following siRNA knockdown, 75K MDA-MB-231-4175 TNBC cells were plated on Matrigel in 48-well plates, and tube formation was monitored in the Incucyte (Essen), with images acquired every hour for 16 hours. Trajectory plots, accumulated distance, Euclidean distance, and velocity of tumor cells as they migrated on matrigel were calculated using the Chemotaxis and Migration Tool (Ibidi).

### *In Vivo* Orthotopic TNBC Xenograft Model

To assess changes in the markers of interest observed *in vitro*, 5 × 10^6^ MDA-MB-231-4175 lung tropic tumor cells were orthotopically implanted into the mammary fat pad of female NOD scid gamma (NSG) mice. Mice were randomized into treatment groups and treated 2x weekly, intravenously, starting on day 3, with PBS, E-P125A, cetuximab, or αEGFR-E-P125A. Mice were sacrificed on day 26, and primary tumors and lung tissues were collected, weighed, and flash frozen for further downstream analysis. Lung tissues were harvested and stained with a human cytokeratin cocktail to detect tumor cell infiltration [BD Cytokeratin (catalog no. 349205), Dako Cytokeratin AE1/AE3 (catalog no. M351504-2), and Dako Cytokeratin HMK (catalog no. M063001-2)], and primary tumors were stained for mCD31 (catalog no. sc-376764 AF546) and huLaminin (catalog no. sc-13586 AF488) as described in our previous study (12).

### Cancer Dependency (DepMap) Portal and GEPIA2 Analysis

The Cancer Dependency Map database (DepMap 2022) was used to reference gene expression data acquired from multiple breast cancer cell lines to survey the expression levels of genes of interest, such as *PTK2*, *EGFR*, *ITGA5*, *ITGB1*, and *STAT3*, in TNBC subtypes compared with non-TNBC subtypes. The Gene Expression Profiling Interactive Analysis (GEPIA2 2023) database was used to access public genomic data on patient-derived breast tumors to survey the expression levels of the same genes of interest in patients with TNBC subtypes compared with non-TNBC subtypes.

### Statistical Analysis

Data are expressed as mean ± SEM from three or more independent experiments. Student *t* test was used for the comparisons between two groups, and one-way ANOVA was used to compare more than two groups. All analyses were performed using GraphPad Prism (Version 9.5, GraphPad Software Inc.). Statistical significance was set at *P* value < 0.05.

### Data Availability

The RNA-seq data generated in this study were deposited into the Gene Expression Omnibus under the accession number GSE253789 for public availability. The Hallmarks gene sets (h.all.v2022.1.Hs.symbols.gmt) for GSEA were accessed from the GSEA website (https://www.gsea-msigdb.org/gsea/msigdb/). Additional data generated and computational methods used in this study are available upon request.

### Ethical Compliance

All procedures performed in studies involving animal models were conducted ethically and approved by the University of Miami Miller School of Medicine (Miami, FL) and the Institutional Animal Care and Use Committee.

## Results

### Elucidating the Molecular Signaling Mechanisms of αEGFR-E-P125A Inhibition

**FIGURE 1:**
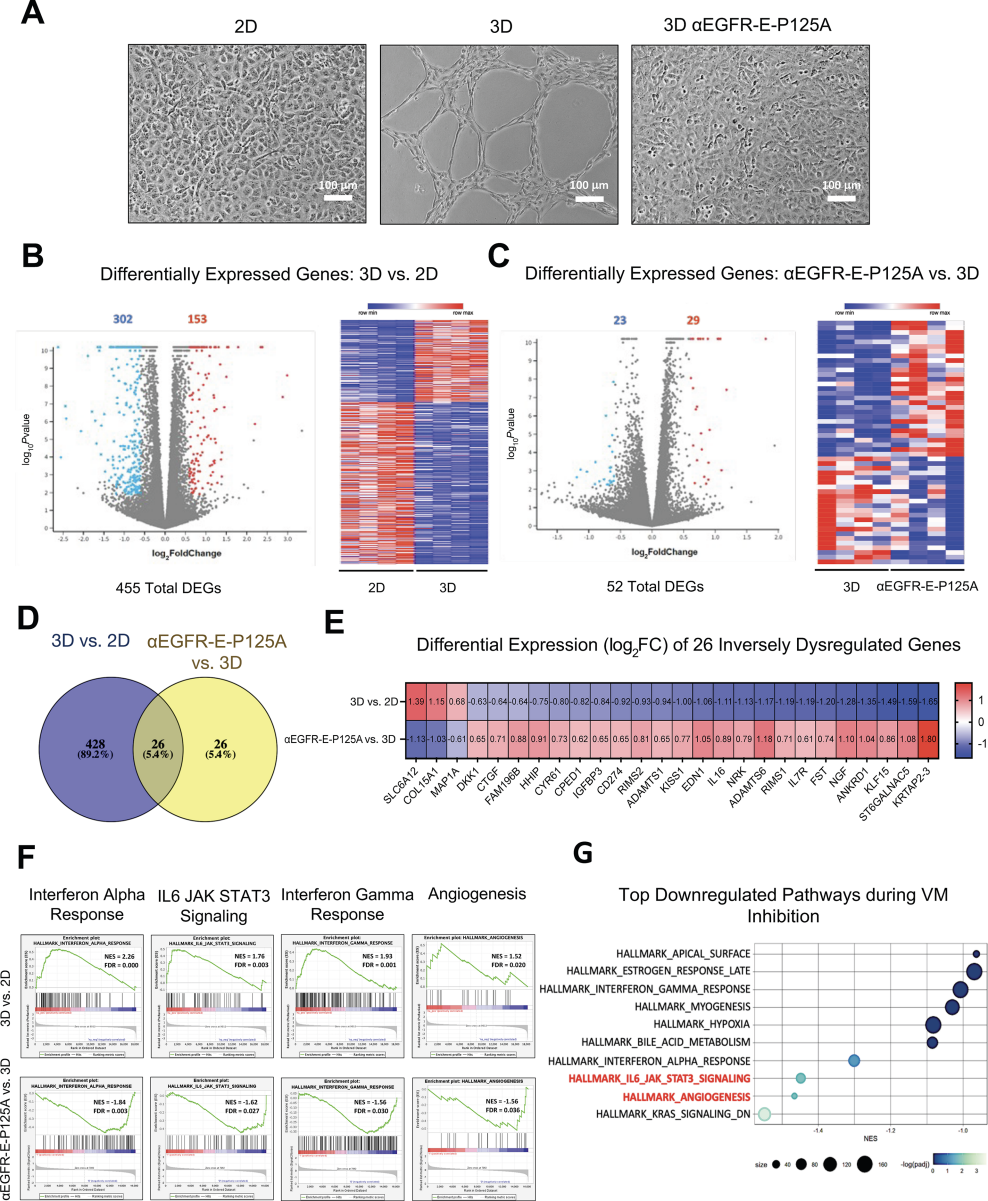
Elucidating the molecular signaling mechanisms of αEGFR-E-P125A inhibition. **A,** MDA-MB-231-4175 cells plated in a monolayer (2D), on matrigel (3D), or on matrigel and treated with αEGFR-E-P125A (3D αEGFR-E-P125A). **B,** Volcano plot depicts 455 total DEGs between 2D and 3D conditions. A total of 153 genes were upregulated while 302 genes were downregulated upon the 2D to 3D transition (left). Heat map depicts the top 100 DEGs between 2D and 3D conditions based on z-score. **C,** Volcano plot depicts 52 DEGs between 3D and αEGFR-E-P125A conditions. Twenty-nine genes were upregulated while 23 genes were downregulated upon the 3D to αEGFR-E-P125A transition. Heat map depicts all the DEGs from 3D to αEGFR-E-P125A–treated transition based on z-score (right). **D,** Venn diagram representing the 26 genes from the each of the differentially expressed lists which were identified as being inversely dysregulated upon the induction of VM by matrigel and the inhibition of VM by αEGFR-E-P125A treatment. **E,** Heat map depicting the log_2_FC of the 26 inversely dysregulated genes from 2D to 3D and from 3D to αEGFR-E-P125A treatment transitions. **F,** GSEA using the Hallmarks gene set depicts inversely related pathways which were upregulated from 2D to 3D and downregulated upon the 3D to αEGFR-E-P125A treatment transition. **G,** On the basis of the pathways which were identified as being upregulated from 2D to 3D and inversely downregulated from 3D to αEGFR-E-P125A treatment conditions, the top downregulated pathways identified were the IL6-JAK-STAT3 and the angiogenesis signaling pathways.

Although VM has been associated with various oncogenic signaling pathways, the transcriptional changes responsible for regulating the induction and inhibition of VM in tumor cells are poorly understood. To identify genes associated with the induction and/or inhibition of VM in TNBC, RNA-seq was conducted on MDA-MB-231-4175 TNBC cells grown in a monolayer and not engaging in VM (2D), grown on Matrigel and leading to VM (3D), or TNBC cells grown on Matrigel and treated with αEGFR-E-P125A with VM inhibited (αEGFR-E-P125A; Fig. 1A; Supplementary Fig. S1; Supplementary Table S1). Differential gene expression analysis comparing MDA-MB-231-4175 samples plated in 2D with cells plated in 3D was performed and demonstrated that the transition from 2D to 3D identified a total of 455 DEGs. A total of 153 genes were upregulated and 302 genes were downregulated upon the induction of VM. A volcano plot and heat map were used to depict the top DEGs identified in the 2D to 3D transition (Fig. 1B; Supplementary Fig. S2; Supplementary Table S2). When cells in 3D were compared with TNBC cells plated in 3D and treated with αEGFR-E-P125A, RNA-seq analysis identified a total of 52 DEGs. Twenty-nine of the 52 DEGs were transcriptionally upregulated relative to the 3D control, while the 23 remaining genes were transcriptionally downregulated upon treatment with αEGFR-E-P125A (Fig. 1C). A total of 26 genes were inversely regulated during the induction of VM (2D to 3D transition) compared with the inhibition of VM in the 3D-to-3D αEGFR-E-P125A–treated transition (Fig. 1D).

A heat map was constructed for the 26 common genes that were identified within both transitions and demonstrated the log_2_FC of each of the inversely DEGs (Fig. 1E). As a result of few DEGs, pathways analysis could not identify a set of dysregulated signaling pathways using the DEGs alone. However, dysregulation of these genes alone may be the minimal requirement for the inhibition of VM using αEGFR-E-P125A. While each of the DEGs may play a role in the formation of VM independently, they were not all closely related and did not fall within a single signaling pathway. The aggregate effect of multiple subtle changes occurring along signaling pathways was studied using GSEA. GSEA using Hallmarks datasets was used to identify the pathways upregulated during VM induction (3D vs. 2D) and downregulated by αEGFR-E-P125A treatment (αEGFR-E-P125A vs. 3D). The key pathways identified included: interferon alpha response, IL6-JAK-STAT3 signaling, IFN gamma response, and the angiogenesis signaling pathway (Fig. 1F). Two key pathways that were identified, the angiogenesis and IL6-JAK-STAT3 signaling pathways, were upregulated upon VM induction and inversely downregulated upon VM inhibition by αEGFR-E-P125A treatment (Fig. 1G). GSEA using the Hallmarks gene sets also identified several key pathways that were downregulated from 2D to 3D induction of VM and inversely upregulated from 3D to αEGFR-E-P125A treatment and VM inhibition. The identified pathways included Myc targets V2, E2F targets, and the G_2_–M checkpoint (Supplementary Fig. S3A and S3B).

### Mechanism of αEGFR-E-P125A Inhibition of VM in the Transcriptome

**FIGURE 2:**
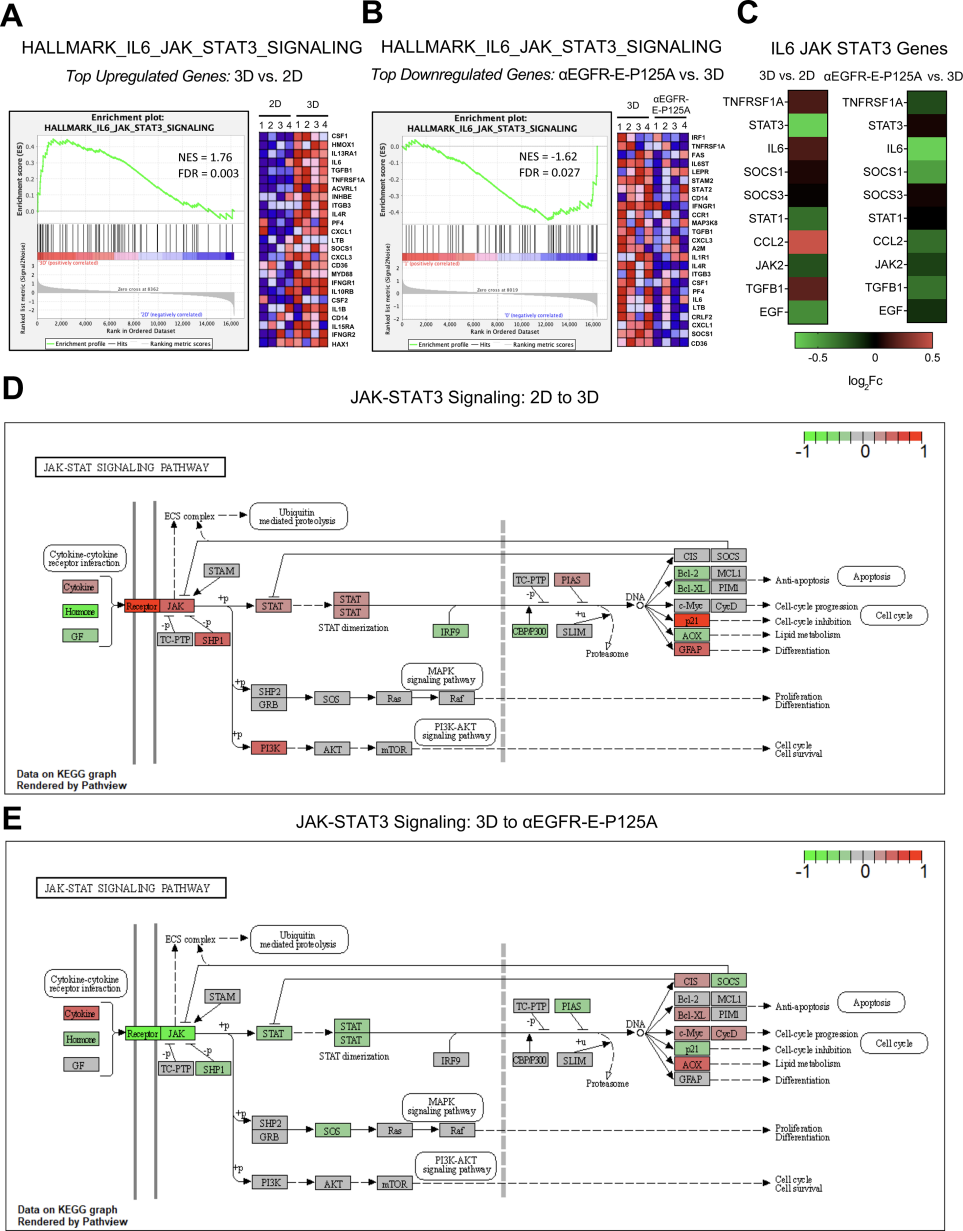

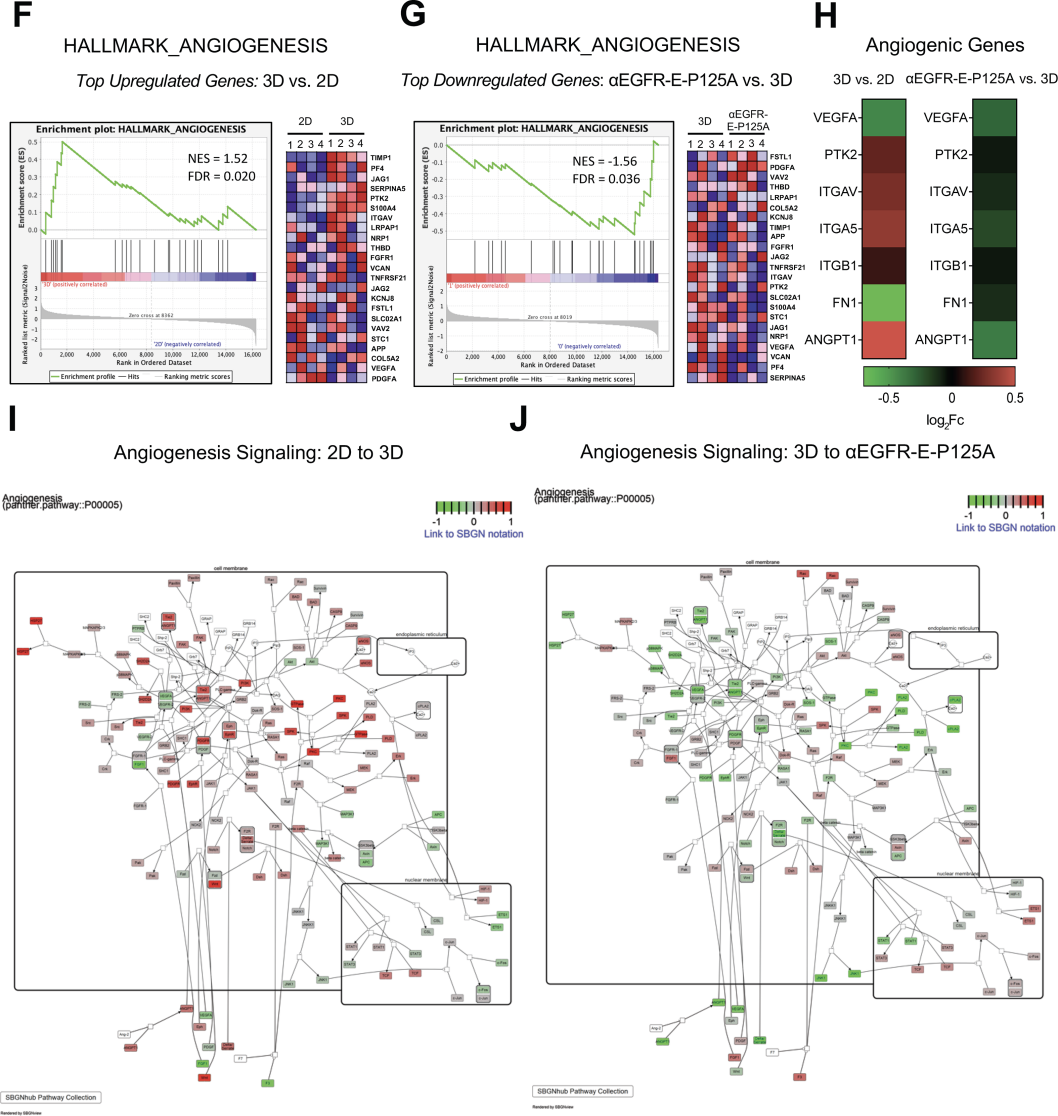
Mechanism of αEGFR-E-P125A action on inhibition of VM in the transcriptome. **A,** As previously shown, genes along the IL6-JAK-STAT3 signaling pathway are significantly upregulated from 2D to 3D using GSEA enrichment plot (left), with top upregulated genes along pathway depicted on the heat map (right). **B,** Genes along the IL6-JAK-STAT3 signaling pathway are significantly downregulated from 3D to the 3D αEGFR-E-P125A treatment transition using GSEA (left), with top downregulated genes depicted on the heat map (right). **C,** NanoString validation of RNA-seq data demonstrates that many JAK-STAT3 signaling genes are upregulated from 2D to 3D (left) and then downregulated upon αEGFR-E-P125A treatment (right). **D,** Integrated Pathview plot derived from KEGG database demonstrates upregulation of specific genes of interest within the JAK-STAT3 signaling pathway from the 2D to 3D transition. **E,** Pathview plot demonstrates overall inverse downregulation of JAK-STAT3 signaling pathway from the 3D-to-3D αEGFR-E-P125A treatment transition. **F,** As shown previously, genes along the Angiogenesis signaling pathway are significantly upregulated from 2D to 3D using GSEA enrichment plot (left), with top upregulated genes along pathway depicted on the heat map (right). **G,** Genes along the angiogenesis signaling pathway are significantly downregulated from 3D to the 3D αEGFR-E-P125A treatment transition using GSEA (left), with top downregulated genes depicted on the heat map (right). **H,** NanoString validation of RNA-seq data demonstrates that many angiogenesis signaling genes are upregulated from 2D to 3D (left) and then downregulated upon αEGFR-E-P125A treatment (right). **I,** Integrated SBGNview plot derived from Panther database demonstrates upregulation of genes of interest within the angiogenesis signaling pathway from 2D to 3D. **J,** SBGNview plot demonstrates overall downregulation of the angiogenesis signaling pathway upon 3D to αEGFR-E-P125A treatment transition.

The IL6-JAK-STAT3 signaling cascade in TNBC is known to regulate angiogenesis, tumor cell invasion, metastasis, and therapeutic resistance in TNBC (32). Blocking IL6-JAK-STAT3 signaling has been shown to promote TNBC chemosensitivity (33). As noted previously, GSEA demonstrated that the IL6-JAK-STAT3 signaling pathway was significantly upregulated upon the induction of VM (Fig. 2A) and downregulated upon the inhibition of VM by αEGFR-E-P125A (Fig. 2B). NanoString validation of RNA-seq data confirmed the downregulation of multiple genes associated with the JAK-STAT3 signaling pathway upon αEGFR-E-P125A treatment including *TNFRSF1A*, *IL6*, *SOCS1*, *CCL2*, *JAK2*, *TGFB1*, and *EGF* (Fig. 2C). Integrated pathway analysis using the Kyoto Encyclopedia of Genes and Genomes (KEGG) database demonstrated overall upregulation of multiple genes along the JAK-STAT3 signaling pathway upon the induction of VM in 3D (Fig. 2D) and downregulation of JAK and STAT genes, consistent with the overall downregulation of the JAK-STAT3 signaling pathway upon treatment with αEGFR-E-P125A (Fig. 2E).

A second downregulated pathway identified upon αEGFR-E-P125A treatment was the Hallmarks angiogenesis signaling pathway. Tumor angiogenesis is known to be regulated by VEGFA and VEGF receptors, such as VEGFR2, in TNBC subtypes (34). In addition to regulation by VEGFs, tumor angiogenesis in TNBC is reported to be regulated in part through FAK-mediated promotion of VEGFR-2 expression, and targeting FAK has been proposed as an antiangiogenic strategy for the treatment of TNBC (35). As shown in Fig. 1, GSEA demonstrated that 3D VM induction led to an increase in angiogenesis signaling (Fig. 2F), while αEGFR-E-P125A treatment led to the downregulation of genes in the angiogenesis signaling pathway (Fig. 2G). NanoString validation of angiogenic RNA-seq data demonstrated downregulation of genes associated with angiogenesis, including *VEGFA*, *PTK2* (FAK), *ITGAV*, *ITGA5*, *FN1*, and *ANGPT1* upon αEGFR-E-P125A treatment (Fig. 2H). Integrated pathway analysis using the Panther database demonstrated upregulation of angiogenesis signaling pathway genes during the induction of VM (Fig. 2I) and an overall downregulation of genes along the angiogenesis signaling network upon αEGFR-E-P125A treatment, including downregulation of genes encoding various known angiogenic regulators, including *TIE2*, *ANGPT1*, *FAK*, and *VEGFA* (Fig. 2J). Together, these results indicate that there is an overall transcriptional downregulation of genes related to JAK-STAT3 and angiogenesis signaling, which is associated with the abrogation of VM in αEGFR-E-P125A–treated tumor cells.

### αEGFR-E-P125A Downregulates EGFR, FAK, and STAT3 Phosphorylation

**FIGURE 3:**
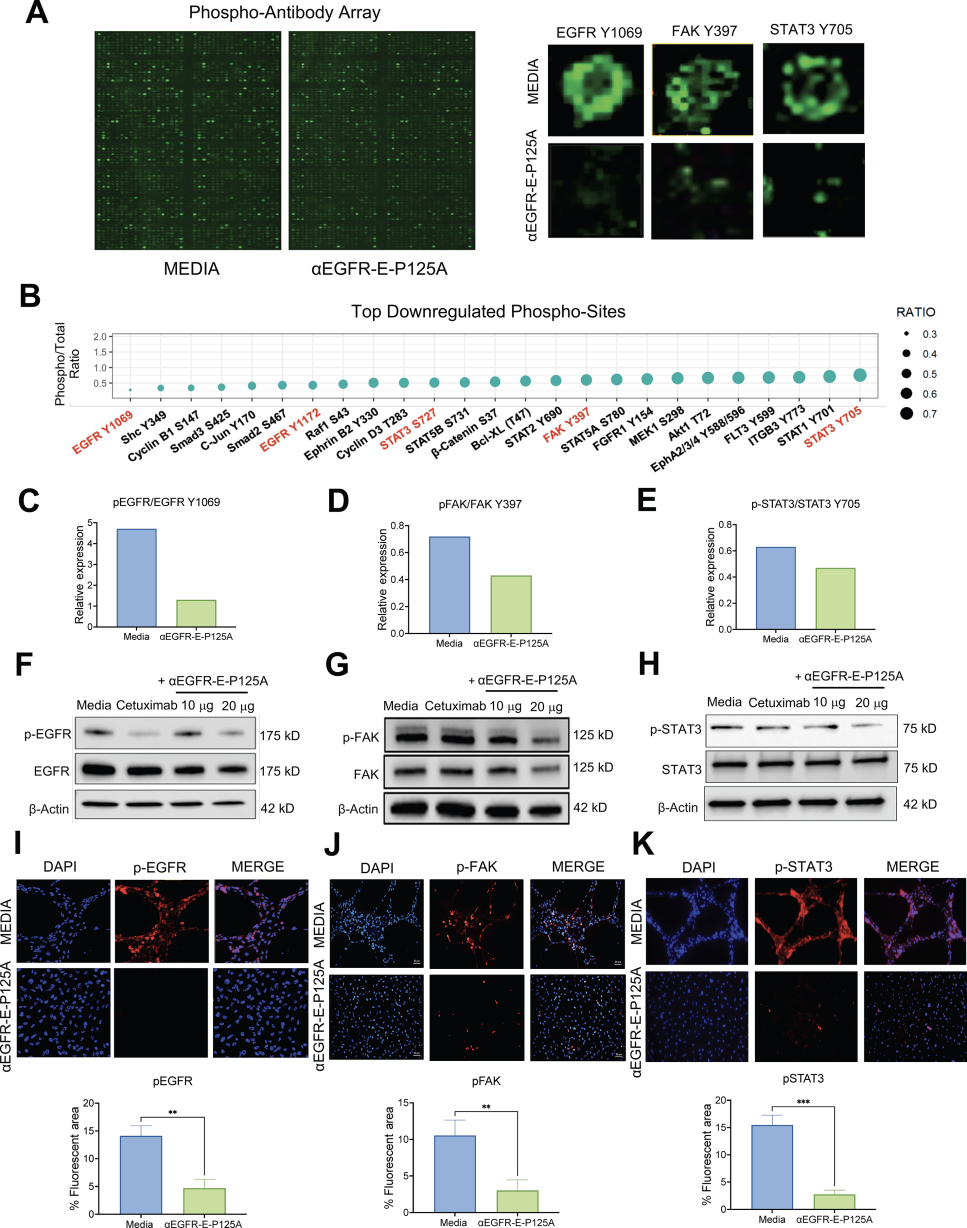
αEGFR-E-P125A inhibits EGFR, FAK, and STAT3 phosphorylation. **A,** Phospho-antibody array depicts changes in phosphorylation from MDA-MB-231-4175 cells plated on matrigel and undergoing the tube formation process to those plated on matrigel and treated with αEGFR-E-P125A for 16 hours in which tube formation was inhibited (left). Zoomed panel depicts decreased EGFR Y1069, FAK Y397, and STAT3 Y705 phosphorylation upon αEGFR-E-P125A treatment (right). **B,** Bubble plot depicts top downregulated phospho-sites as seen in the phospho-antibody array. Downregulated phospho-sites of interest which correlate to transcriptomic findings include EGFR Y1069, FAK Y397, and STAT3 Y705 sites. **C–E,** Quantification of EGFR Y1069, FAK Y397, and STAT3 Y705 downregulation from phospho-array. **F–H,** Western blots demonstrating downregulation of EGFR Y1069, FAK Y397, and STAT3 Y705 upon αEGFR-E-P125A treatment compared with controls. **I–K,** Immunofluorescence staining at 20x magnification demonstrating inhibition of tube formation and downregulation of EGFR Y1069, FAK Y397, and STAT3 Y705 upon αEGFR-E-P125A treatment. Staining was quantified by percent fluorescent area. **, *P* < 0.01; ***, *P* < 0.001.

In addition to changes in the transcriptome, we investigated the signaling changes in the phospho-kinome that occurred with αEGFR-E-P125A treatment in 3D. Phospho-antibody array analysis demonstrated that the treatment of MDA-MB-231-4175 cells with αEGFR-E-P125A downregulated various phosphorylation sites, including the phosphorylation of EGFR at tyrosine 1069 (Y1069), FAK at tyrosine 397 (Y397), and STAT3 at tyrosine 705 (Y705) (Fig. 3A–E). Phosphorylation of EGFR at Y1069 occurs upon dimerization of EGFR and is a site of aberrant EGFR signaling, which promotes TNBC motility (36). FAK phosphorylation at the Y397 site occurs downstream of β1 and β3 integrins and can additionally occur following EGFR-mediated Src activation (37). FAK phosphorylation at Y397 has been implicated in the regulation of endothelial and TNBC motility, VM, and metastatic behavior (38, 39). Phosphorylation of STAT3 at Y705 in tumor cells is known to promote the transcription of angiogenic genes such as *TGFβ, VEGF, HIF1α, MMP2/9, Cyclin D1, c-MYC*, and *IL6* (40). Protein expression levels of p-EGFR Y1069 decreased with both cetuximab and αEGFR-E-P125A treatment relative to the control, whereas p-FAK Y397 and p-STAT3 Y705 decreased in a dose-dependent manner upon treatment with αEGFR-E-P125A but not with an equimolar concentration of cetuximab (Fig. 3F–H). The reduced levels of p-EGFR, p-FAK, and p-STAT3 at the aforementioned phosphorylation sites were further verified by immunofluorescence staining (Fig. 3I–K). These findings suggest that αEGFR-E-P125A treatment may inhibit VM through reduced phosphorylation and activation of EGFR, FAK, STAT3, and their associated signaling pathways.

### αEGFR-E-P125A Suppresses α5 Integrin and EGFR Activation, and Consequently Downregulates FAK/STAT3 Signaling

**FIGURE 4:**
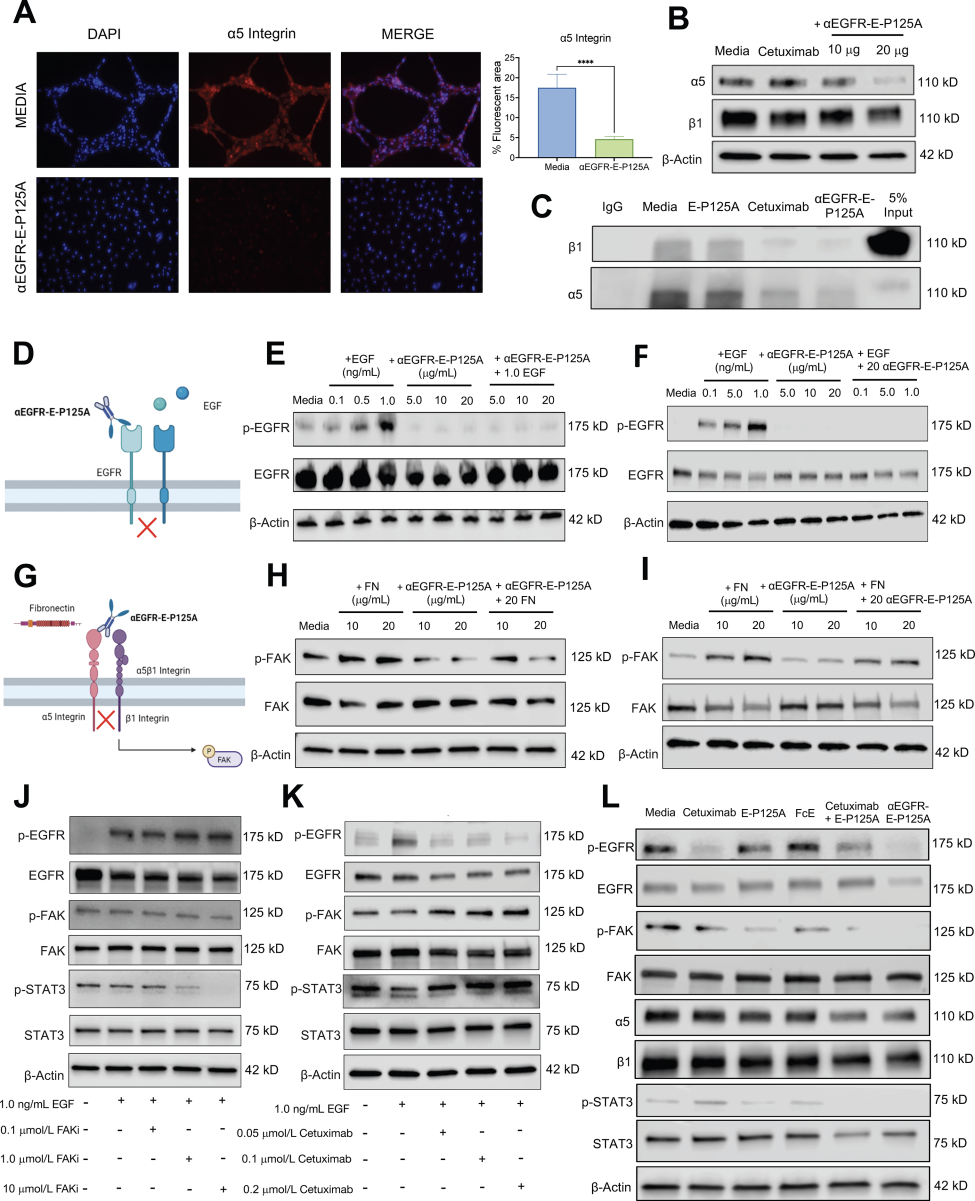
αEGFR-E-P125A suppresses α5 integrin and EGFR activation, and consequently downregulates FAK/STAT3 signaling. **A,** Immunofluorescence staining at the 20x magnification demonstrates that expression of α5 integrin is disrupted upon treatment with αEGFR-E-P125A. Staining was quantified by percent fluorescent area. **B,** Western blot analysis of MDA-MB-231-4175 cells plated on matrigel and treatment with either controls or αEGFR-E-P125A. Western blot analysis demonstrates downregulation of α5 integrin upon αEGFR-E-P125A treatment compared with controls. **C,** IP of α5 integrin was conducted on samples treated with an IgG control, media, E-P125A, cetuximab, or αEGFR-E-P125A. IP demonstrated that cells treated with αEGFR-E-P125A had decreased levels of α5 integrin after pulldown, resulting in decreased levels of β1 integrin bound to α5 integrin compared with the controls. **D,** Schematic demonstrating αEGFR-E-P125A binding to EGFR via the α-EGFR antibody end and preventing EGF-induced EGFR phosphorylation at EGFR Y1069. **E,** EGFR competition experiment between αEGFR-E-P125A and EGF ligands. MDA-MB-231-4175 cells were either stimulated with EGF alone, treated with αEGFR-E-P125A, or pretreated with αEGFR-E-P125A and then stimulated with EGF. **F,** Reverse EGFR competition experiment between αEGFR-E-P125A and EGF ligands. MDA-MB-231-4175 cells were pretreated with either stimulated with EGF alone, treated with αEGFR-E-P125A, or prestimulated with EGF and then treated with αEGFR-E-P125A. **G,** Schematic demonstrating αEGFR-E-P125A binding to α5β1 integrin via the E-P125A end and preventing fibronectin-induced FAK Y397 phosphorylation. **H,** α5β1 integrin competition experiment between αEGFR-E-P125A and fibronectin. MDA-MB-231-4175 cells were either stimulated with fibronectin alone, treated with αEGFR-E-P125A, or pretreated with αEGFR-E-P125A and then stimulated with fibronectin. **I,** Reverse α5β1 integrin competition experiment between αEGFR-E-P125A and fibronectin. MDA-MB-231-4175 cells were either stimulated with fibronectin alone, treated with αEGFR-E-P125A, or prestimulated with fibronectin and then treated with αEGFR-E-P125A. **J,** Treatment of MDA-MB-231-4175 cells with increasing concentrations of FAK inhibitor, PF-573,228 demonstrated decrease in FAK Y397 and downstream STAT3 Y705 phosphorylation, and a subsequent increase in EGFR Y1069 phosphorylation. **K,** Treatment of MDA-MB-231-4175 cells with increasing concentrations of cetuximab demonstrated decrease in EGFR Y1069 and total EGFR levels, and a subsequent increase in FAK Y397 and STAT3 Y705 phosphorylation. **L,** Treatment of MDA-MB-231-4175 cells with either media, cetuximab, E-P125A, the untargeted FcE dimer, a combination of cetuximab and E-P125A, or αEGFR-E-P125A for 16 hours in 3D demonstrated enhanced inhibition of downstream EGFR and α5β1 integrin signaling upon treatment with αEGFR-E-P125A.

Downstream phosphorylation of FAK and STAT3 can be regulated by EGFR signaling or by α5β1 integrin activation through the binding of fibronectin (23, 41, 42). We demonstrated a reduction in FAK phosphorylation at Y397, a site known to be regulated by α5β1 integrin. Endostatin is a known ligand for α5β1. Using immunofluorescence, we analyzed the effects of αEGFR-E-P125A on α5 integrin protein levels in MDA-MB-231-4175 cells. Immunofluorescence staining demonstrated a decrease in the percent area fluorescence of α5 integrin protein upon αEGFR-E-P125A treatment (Fig. 4A). Western blot analysis of MDA-MB-231-4175 cells plated on Matrigel and treated with αEGFR-E-P125A for 16 hours revealed a dose-dependent reduction in α5 integrin protein levels (Fig. 4B). Because the α5 integrin subunit uniquely colocalizes with the β1 integrin subunit to promote downstream signaling and the subsequent phosphorylation and activation of FAK, we performed IP for the α5 integrin subunit posttreatment with αEGFR-E-P125A and found a reduction in β1 integrin coprecipitation with α5 integrin following treatment with αEGFR-E-P125A (Fig. 4C).

We next tested whether αEGFR-E-P125A prevents prospective ligands from binding to and activating EGFR and α5β1 integrin receptors, respectively. EGF is the primary ligand that binds to EGFR and promotes its receptor dimerization and activation, resulting in EGFR phosphorylation (43). We tested whether αEGFR-E-P125A prevented EGF-induced EGFR phosphorylation at Y1069. MDA-MB-231-4175 cells were either pretreated for 2 hours with αEGFR-E-P125A followed by stimulation with EGF, or prestimulated with EGF for 30 minutes followed by αEGFR-E-P125A treatment. In both cases, EGF-induced phosphorylation of EGFR Y1069 was inhibited by αEGFR-E-P125A (Fig. 4D–F).

Fibronectin binds to α5β1 integrin via the RGD binding site and promotes a conformational change that leads to downstream phosphorylation and activation of FAK at Y397 (44). Studies have suggested that endostatin also binds to α5β1 integrin in an RGD motif-dependent manner and may compete for the fibronectin binding site (45). Therefore, we tested whether αEGFR-E-P125A can prevent fibronectin-induced FAK phosphorylation at Y397. MDA-MB-231-4175 cells were either pretreated with αEGFR-E-P125A for 2 hours followed by stimulation with fibronectin, or prestimulated with fibronectin for 2 hours followed by treatment with αEGFR-E-P125A (Fig. 4G). MDA-MB-231-4175 cells pretreated with αEGFR-E-P125A and then stimulated with fibronectin demonstrated reduced fibronectin-induced FAK phosphorylation at Y397 (Fig. 4H). In contrast, when cells were prestimulated with fibronectin and treated with αEGFR-E-P125A, there was no change in the levels of fibronectin-induced FAK phosphorylation at Y397, suggesting that αEGFR-E-P125A and fibronectin may competitively bind α5β1 integrin (Fig. 4I).

Studies have demonstrated that integrins such as α5β1 and growth factor receptors such as EGFR may compensate for one another and promote resistance to therapies targeting either receptor alone (29). To better understand the cross-talk occurring downstream of α5β1 integrin and EGFR respectively, we independently inhibited the p-FAK Y397 and p-EGFR Y1069 sites and observed downstream signaling events. We used increasing doses of the small-molecule FAK inhibitor, PF-573,228 (Sigma-Aldrich, catalog no. PZ0117), which specifically inhibits the phosphorylation of FAK at the Y397 site (46). Treatment with PF-573,228 demonstrated that decreased FAK phosphorylation at Y397 was associated with reduced STAT3 phosphorylation at Y705, but increased EGFR phosphorylation at Y1069 (Fig. 4J). Conversely, treatment of MDA-MB-231-4175 cells with increasing doses of cetuximab, resulted in decreased levels of phosphorylated EGFR Y1069, accompanied by an increase in FAK Y397 and STAT3 Y705 phosphorylation levels (Fig. 4K). Therefore, inhibition of either p-FAK Y397 or p-EGFR Y1069 alone leads to the compensatory phosphorylation of the other protein.

It has been shown that targeting integrins can overcome the acquired resistance and sensitize growth factor–driven cancers to treatments with mAbs (47). Western blot analysis of MDA-MB-231-4175 cells treated in 3D with either cetuximab, E-P125A, the untargeted Fc-Endostatin (FcE) dimer, a combination of cetuximab and E-P125A, or αEGFR-E-P125A for 16 hours demonstrated that the combination of cetuximab and E-P125A modestly decreased phosphorylation of p-EGFR Y1069, p-FAK Y397, p-STAT3 Y705, and α5 integrin levels, while αEGFR-E-P125A treatment more effectively reduced levels of these proteins compared with the controls (Fig. 4L). A time-course analysis of MDA-MB-231-4175 cells treated with αEGFR-E-P125A in 2D confirmed a similar reduction in both the EGFR and α5β1 integrin/FAK signaling proteins at early timepoints in the 2D environment (Supplementary Fig. S4A). The simultaneous suppression of both EGFR and α5β1 integrin signaling by αEGFR-E-P125A may be useful in overcoming therapeutic resistance.

### Inhibition of EGFR Signaling is not Critical for Inhibition of VM by αEGFR-E-P125A

**FIGURE 5:**
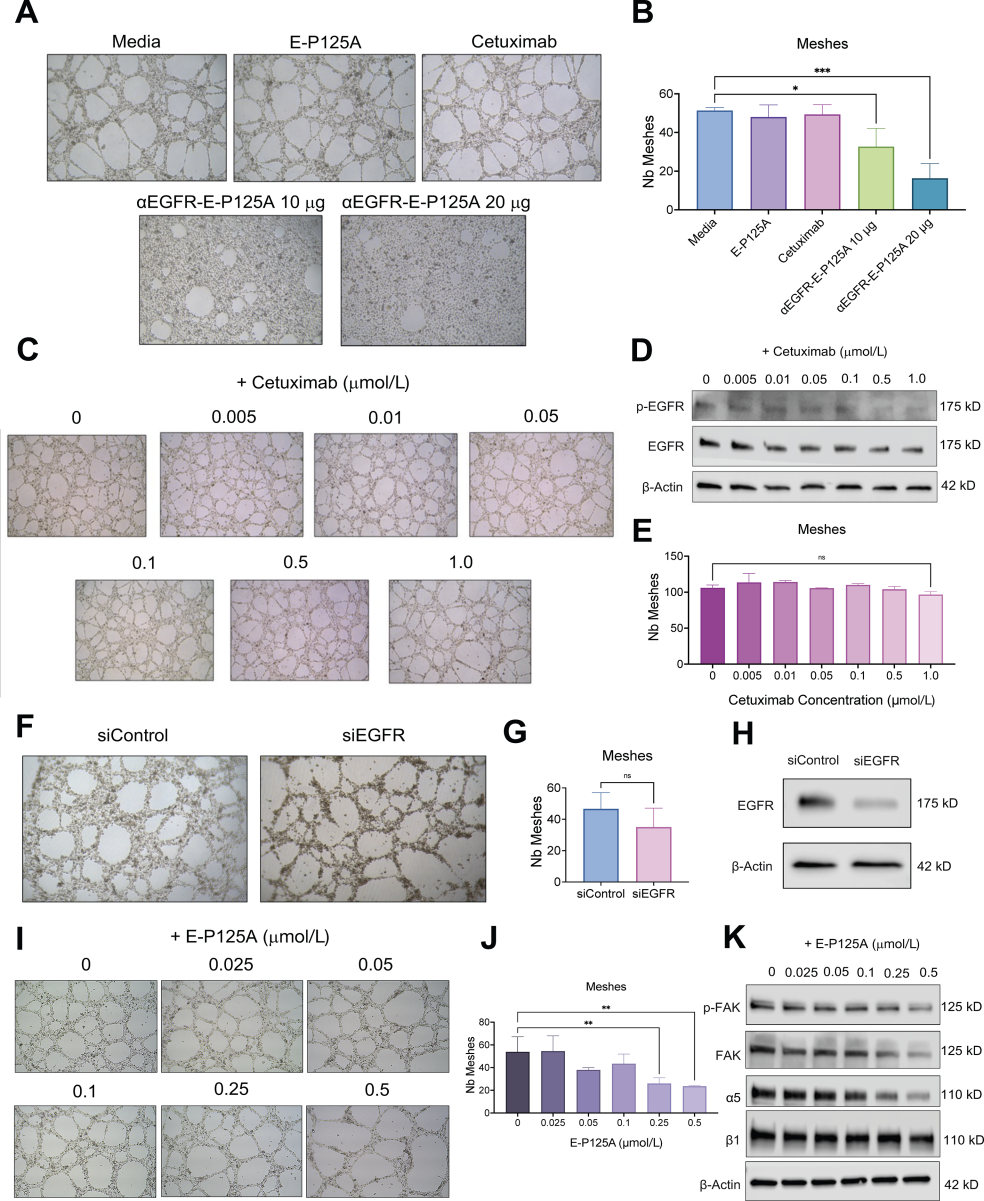
Inhibition of EGFR signaling is not critical for inhibition of VM by αEGFR-E-P125A. **A,** αEGFR-E-P125A treatment inhibits VM tube formation in a dose-dependent manner compared with cetuximab and E-P125A controls. **B,** Quantification of meshes by Image J Angiogenesis Analyzer plugin demonstrates that compared with controls, αEGFR-E-P125A treatment significantly reduced mesh (tube) formation in MDA-MB-231-4175 cell plated on matrigel. **C,** Increasing doses of cetuximab treatment does not inhibit VM tube formation. **D,** Western blot analysis demonstrating reduction of EGFR Y1069 phosphorylation upon increasing doses of cetuximab treatment. **E,** Quantification of meshes by Image J Angiogenesis Analyzer plugin demonstrates that increasing doses of cetuximab do not inhibit VM tube formation. **F,** siRNA knockdown of EGFR was conducted on MDA-MB-231-4175 cells and a VM tube formation assay was conducted to determine whether knockdown of EGFR inhibited VM tube formation. **G,** Quantification of meshes demonstrates that number of meshes remained the same upon EGFR knockdown. **H,** Western blot analysis validating efficiency of EGFR siRNA knockdown. **I,** Increasing doses of E-P125A results in the formation of fewer VM tubes. **J,** Quantification of meshes demonstrates that increasing concentrations of E-P125A reduces number of complete meshes. **K,** Western blot analysis demonstrating reduction of FAK Y397 phosphorylation upon increasing concentrations of E-P125A treatment. VM tube formation assays were imaged at 5x magnification. ns, not significant; *, *P* < 0.05; **, *P* < 0.01; ***, *P* < 0.001.

αEGFR-E-P125A treatment inhibits VM in a dose-dependent manner. However, when MDA-MB-231-4175 cells were treated with either E-P125A or cetuximab, VM was not inhibited (Fig. 5A). Upon inhibition of VM by αEGFR-E-P125A treatment, the number of meshes (tubes) formed by tumor cells plated on Matrigel decreased (Fig. 5B). To determine whether αEGFR-E-P125A treatment inhibits VM by decreasing the phosphorylation of EGFR at Y1069, we tested the effects of increasing doses of cetuximab on the inhibition of VM and assayed the corresponding levels of p-EGFR. Increasing doses of cetuximab led to a progressive reduction in EGFR Y1069 phosphorylation but had little or no effect on VM tube formation (Fig. 5C and D) or on the number of meshes (Fig. 5E). MDA-MD-231-4175 cells treated with siRNA targeting EGFR demonstrated a reduction in EGFR by Western blotting but did not show reduced VM tube formation (Fig. 5F–H).

To test whether αEGFR-E-P125A treatment inhibited VM by decreasing FAK at Y397, we tested the effects of increasing doses of E-P125A on both VM inhibition and the corresponding levels of p-FAK (Fig. 5I). At higher doses of E-P125A, there was a decrease in the number of meshes formed, in addition to enhanced inhibition of p-FAK at the Y397 site and decreased levels of α5 integrin (Fig. 5J and K). We next tested VM inhibition with the FcE dimer in the absence of EGFR targeting. Although E-P125A demonstrated a modest decrease in tube formation, increasing doses of FcE completely inhibited VM tube formation in a manner similar to that observed with αEGFR-E-P125A treatment (Supplementary Fig. S5A–S5C). FcE delivers a dimeric presentation of E-P125A and demonstrates enhanced inhibition of VM, compared with the E-P125A monomer (48). Our findings indicate that αEGFR-E-P125A VM inhibition is primarily regulated through the effects of dimeric E-P125A on α5β1 integrin/FAK signaling, rather than the inhibition of EGFR signaling.

### α5β1 Integrin/FAK Signaling Regulates VM and Tumor Cell Motility

**FIGURE 6:**
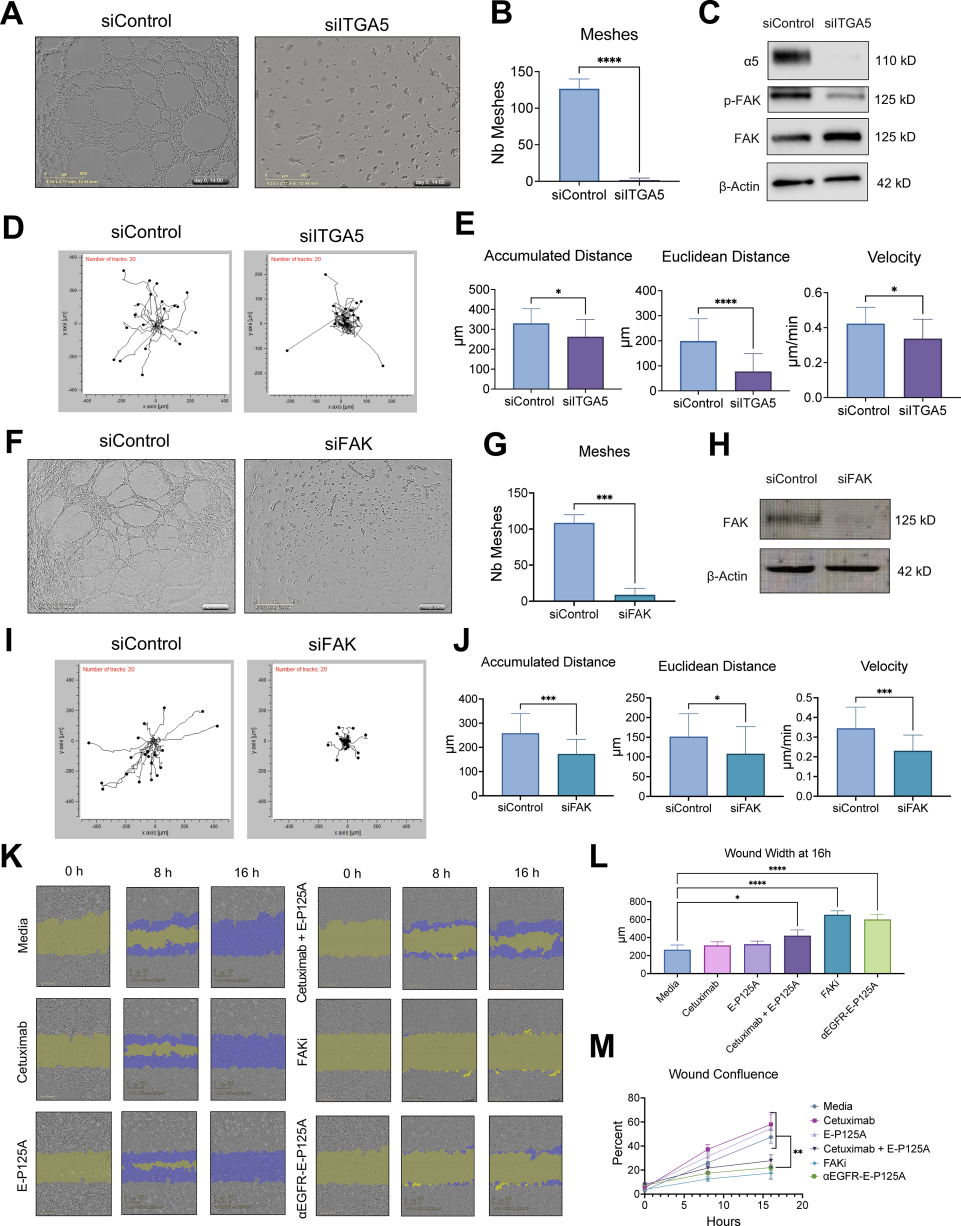
α5β1 integrin/FAK signaling regulates VM and tumor cell motility. **A,** siITGA5 knockdown in MDA-MB-231-4175 cells demonstrates total inhibition of VM tube formation after 16 hours at 4x magnification. **B,** Number of VM meshes significantly decreased upon ITGA5 knockdown. **C,** Western blot validation of siRNA knockdown of ITGA5. Knockdown of ITGA5 subsequently reduces phosphorylation of FAK Y397. **D,** Cell motility tracking demonstrated that ITGA5 knockdown cells had reduced motility and migratory capabilities. **E,** Quantification of motility analysis of ITGA5 knockdown cells demonstrated a reduction in the accumulated and Euclidean distances and decreased velocity. **F,** siFAK knockdown in MDA-MB-231-4175 cells demonstrates total inhibition of VM tube formation after 16 hours at 4x magnification. **G,** Number of VM meshes significantly decreased upon FAK knockdown. **H,** Western blot validation of siRNA knockdown of FAK. **I,** Cell motility tracking demonstrated that FAK knockdown cells had reduced motility and migratory capabilities. **J,** Quantification of motility analysis of FAK knockdown cells demonstrated a reduction in the accumulated and Euclidean distances and decreased velocity. **K,** Incucyte scratch wound migration assay of MDA-MB-231-4175 cells over 16 hours at 10x magnification. Wound closure was partially inhibited in cells treated with a combination of cetuximab of and E-P125A. Wound closure was maximally inhibited in cells treated with either the FAKi, PF-573228, or αE-E-P125A. **L,** Compared to the other controls, the remaining wound width after 16 hours of treatment remained the widest in the FAKi or αEGFR-E-P125A–treated cells. **M,** FAKi and αEGFR-E-P125A treatment had the least changed wound confluence over time. *, *P* < 0.05; **, *P* < 0.01; ***, *P* < 0.001; ****, *P* < 0.0001.

We previously demonstrated a reduction of α5β1 integrin levels and FAK phosphorylation as a result of αEGFR-E-P125A treatment. To directly assess the effects of inactivation of α5β1 integrin on VM tube formation, MDA-MB-231-4175 cells were treated with ATN-161 (Tocris Biosciences, catalog no. 6058), a non-RGD α5β1 integrin antagonist that interacts with the N-terminus of the β1-domain of integrin α5β1 and locks it into an inactive conformation (49). Tumor cells treated with ATN-161 alone demonstrated sustained VM tube formation; however, when combined with cetuximab, there was a reduced number of VM meshes (Supplementary Fig. S6A). Because ATN-161 treatment inactivated the α5β1 integrin conformation**,** while treatment with αEGFR-E-P125A reduced total α5 integrin protein levels, we also tested the effect of siRNA knockdown of α5 integrin (ITGA5). Transient knockdown of ITGA5 reduced VM tube formation and reduced number of quantified meshes (Fig. 6A and B). However, the morphology and distribution of TNBC cells differed markedly from those of αEGFR-E-P125A–treated TNBC. Western blotting confirmed siRNA knockdown of ITGA5, and also demonstrated reduced levels of p-FAK (Fig. 6C). Cell motility analysis demonstrated a reduction in overall motility in siITGA5 cells compared with siControl cells (Fig. 6D), which manifest as decreased accumulated distance traveled, Euclidean distance, and velocity (Fig. 6E).

Because αEGFR-E-P125A treatment reduced FAK phosphorylation at the Y397 site, we also tested the effect of the small-molecule FAK inhibitor, PF-573,228 on VM. Treatment of MDA-MB-231-4175 cells with PF-573,228 resulted in a dose-dependent reduction in VM (Supplementary Fig. S6B and S6C). Next, we used siRNA to reduce total FAK levels. Transient FAK knockdown markedly inhibited VM tube formation (Fig. 6F and G). Western blotting validated siRNA-mediated knockdown of FAK (Fig. 6H). Cell motility analysis demonstrated reduced overall motility in the siFAK cells compared with the siControl cells (Fig. 6I and J). Because reduced FAK phosphorylation correlated with reduced phosphorylation of STAT3 Y705, we next tested the effects of downstream inhibition of JAK/STAT3 phosphorylation using ruxolitinib, a JAK2 inhibitor, or Cryptotanshinone, a JAK2-independent inhibitor of STAT3 Y705 (50). Ruxolitinib-treated TNBC cells also showed reduced VM tube formation when treated alone or in combination with cetuximab (Supplementary Fig. S6D), while Cryptotanshinone inhibition of STAT3 phosphorylation also inhibited VM (Supplementary Fig. S7A and S7C).

Because treatment with αEGFR-E-P125A, as well as knockdown of the genes of interest, inhibited VM and tumor cell motility on Matrigel, we tested the effects of αEGFR-E-P125A treatment on tumor cell motility in a scratch wound assay to assess the effects on motility in a 2D environment. MDA-MB-231-4175 cells were plated in a scratch wound assay and treated with either medium, cetuximab, E-P125A, a combination of cetuximab and E-P125A, the FAKi, (PF-573,228), or αEGFR-E-P125A. Wound healing was monitored over 16 hours and demonstrated that αEGFR-E-P125A treatment inhibited tumor cell wound closure, similar to the inhibition observed in FAKi-treated cells (Fig. 6K). TNBC cells treated with either FAKi or αEGFR-E-P125A had significantly larger wound widths (Fig. 6L) and decreased wound confluence (Fig. 6M) at 16 hours. Our results indicate that inhibition of α5β1 integrin/FAK signaling alone blocks VM tube formation and tumor cell motility.

### αEGFR-E-P125A Reduces Primary Tumor Growth and Lung Metastasis *In Vivo*

**FIGURE 7:**
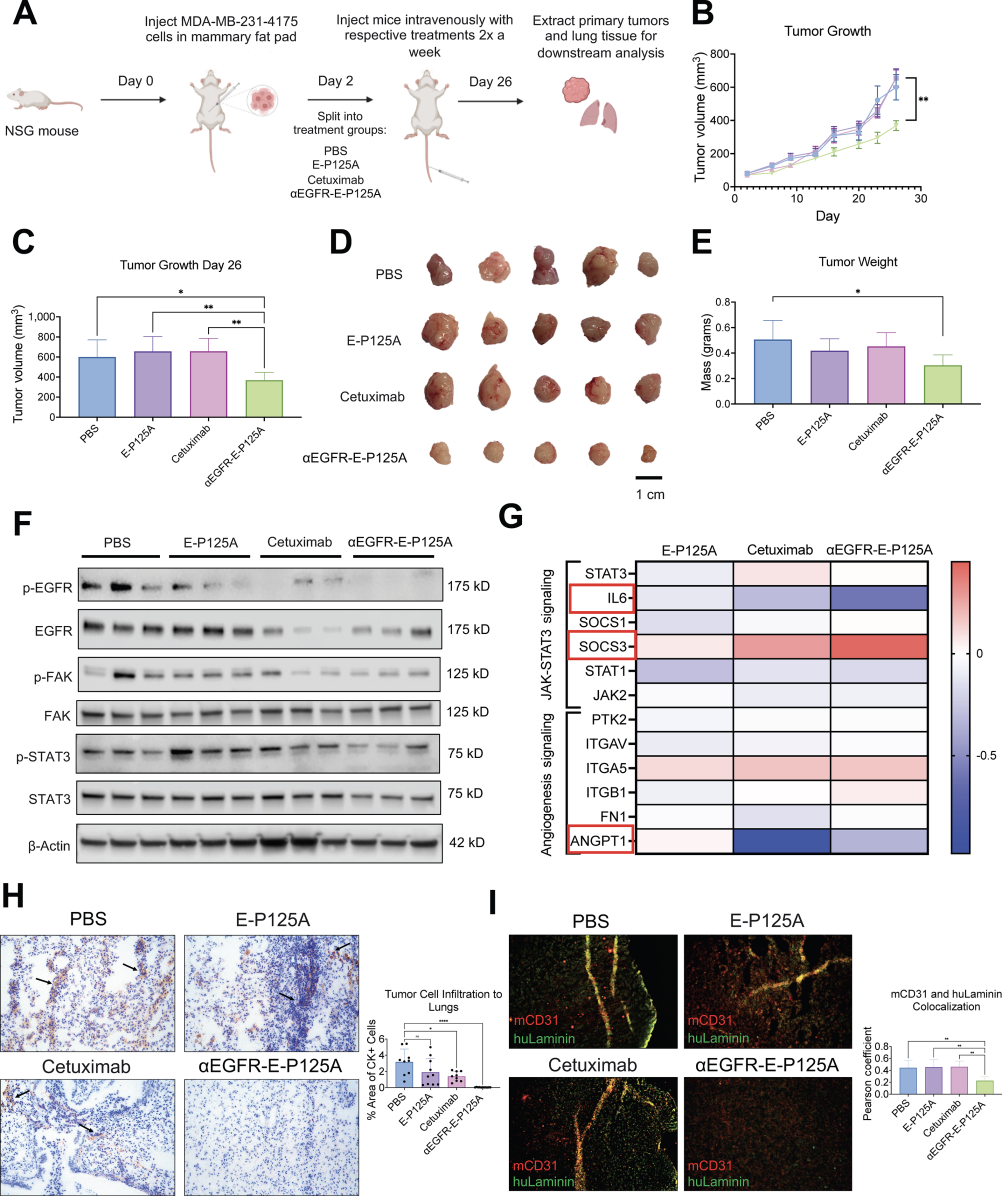
αEGFR-EP125A reduces primary tumor growth and tumor cell infiltration *in vivo*. **A,** Schematic for *in vivo* workflow in NSG mice. MDA-MB-231-4175 TNBC cells were implanted into the mammary fat pad on day 0 and by day 2 tumors had engrafted and mice were randomized and split into four treatment groups: PBS, E-P125A, cetuximab or αEGFR-E-P125A (*n* = 7 mice per group). Mice were treated intravenously with respective treatments 2x a week. By day 26, mice were sacrificed, and tumors and lungs were resected for downstream analysis. **B,** Tumor growth curve demonstrates that mice treated with αEGFR-E-P125A had reduced primary tumor growth compared with mice treated with controls. **C,** Quantification of primary tumor growth at the final day 26 demonstrated that tumors treated with αEGFR-E-P125A were significantly smaller compared with the controls. **D,** Tumor sizes of five representative tumors from each treatment group (*n* = 5 for PBS due to death of 2 mice, and *n* = 7 for E-P125A, cetuximab, and αEGFR-E-P125A groups). **E,** Tumor weight of tumors demonstrated that tumor from mice treated with αEGFR-E-P125A were significantly smaller than the PBS-treated tumors, unlike the controls. **F,** Western blot run on protein extracted from three representative tumor samples demonstrated enhanced downregulation of EGFR Y1069, FAK Y397, and STAT3 Y705 phosphorylation in mice treated with αEGFR-E-P125A compared with the controls. **G,** NanoString validated gene expression changes associated with IL6 JAK STAT3 and Angiogenesis signaling in RNA isolated from *in vivo* tumors (*n* = 3 tumors per group). **H,** Cytokeratin staining of lungs depicts decreased tumor cell infiltration to the lungs at 20x magnification (*n* = 3 tumors per group). **I,** mCD31 staining and huLaminin costaining outlines angiogenic mouse vessels and human VM channels and their respective colocalization within tumors at 20x magnification (*n* = 3 tumors per group). ns, not significant; *, *P* < 0.05; **, *P* < 0.01; ****, *P* < 0.0001.

We next tested whether the signaling effects observed *in vitro* for αEGFR-E-P125A and described above were also observed in MDA-MB-231-4175 TNBC xenografts grown in NSG mice (Fig. 7A). αEGFR-E-P125A–treated tumors were smaller in size, less vascularized (Fig. 7B–D) and had significantly lower tumor volume and weight than control tumors (Fig. 7C–E). Western blot analysis of tumor lysates harvested on day 26 confirmed that αEGFR-E-P125A–treated tumors demonstrated decreased phosphorylation of EGFR at Y1069, FAK at Y397, and STAT3 at Y705 compared with controls (Fig. 7F). As observed *in vitro*, NanoString analysis of RNA isolated from tumors most notably revealed downregulation of *IL6* mRNA, a stimulator of JAK-STAT3 signaling, an increase in *SOCS3*, an inhibitor of STAT3, and decreased levels of *ANGPT1*, which is known to promote angiogenesis (Fig. 7G).

Because MDA-MB-231-4175 TNBC cells are lung-tropic, the lungs from these mice were stained with a human cytokeratin cocktail to assay metastasis. Lungs from mice treated with αEGFR-E-P125A demonstrated significantly reduced spontaneous lung metastasis, as demonstrated by reduced cytokeratin-positive staining compared with control mice (Fig. 7H). Primary tumors acquired from mice were costained for mCD31 and huLaminin to characterize angiogenesis and VM formation, respectively. As shown in Fig. 7I, tumors of mice treated with αEGFR-E-P125A showed reduced mCD31 and huLaminin staining and decreased colocalization *in vivo*. These findings confirm that αEGFR-E-P125A suppression of primary tumor growth and spontaneous lung metastasis is associated with inhibition of angiogenesis, VM, and reduction in IL6-JAK-STAT3 signaling *in vivo*.

### Mechanism of αEGFR-E-P125A Fusion Protein Action

To further assess the possible use of αEGFR-E-P125A as a TNBC therapy, we used the DepMap to determine the expression levels of previously identified markers of interest that were dysregulated upon αEGFR-E-P125A treatment to better understand the relevance of αEGFR-E-P125A as a TNBC therapy. DepMap portal plots using publicly available data derived from human breast cancer cell lines (Supplementary Fig. S8A–S8E) and Gepia2 plots using publicly available tumor samples from patients with breast cancer (Supplementary Fig. S9A–S9E) demonstrated that TNBC subtypes show high expression levels of *PTK2* (FAK), *EGFR*, *ITGA5*, *ITGB1*, and *STAT3*. The increased expression of these markers supports the potential utility of the αEGFR-E-P125A mechanism of action and its potential use as a prospective TNBC targeted therapy.

## Discussion

**FIGURE 8:**
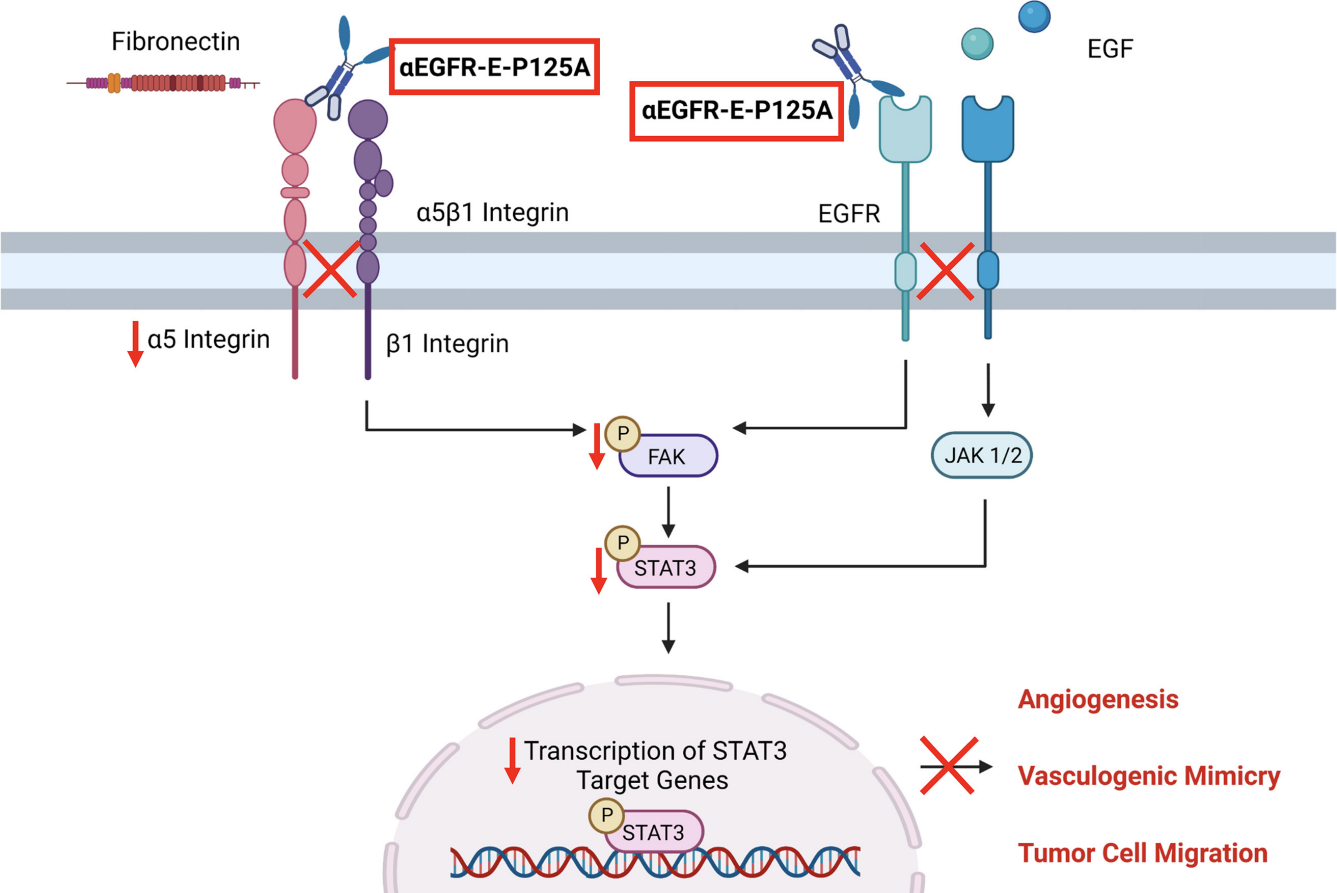
Mechanism of αEGFR-E-P125A fusion protein action. αEGFR-E-P125A inhibits both EGF-induced EGFR phosphorylation and fibronectin-induced FAK phosphorylation mediated through α5β1 integrin signaling, resulting in an overall decrease in FAK phosphorylation at Y397, and STAT3 phosphorylation at Y705. Decreased phosphorylation of STAT3 at Y705 leads to the decreased transcription of STAT3 target genes, leading to decreased angiogenesis, VM, and tumor cell migration and infiltration.

Our studies on the effects of αEGFR-E-P125A action *in vitro* and *in vivo* demonstrated that αEGFR-E-P125A prevents EGF-induced phosphorylation of EGFR at Y1069 and reduces levels of α5β1 integrin, thereby preventing fibronectin-induced phosphorylation of FAK at Y397. Decreased phosphorylation of FAK leads to decreased downstream phosphorylation of STAT3 at the Y705 site, thereby reducing the transcription of certain STAT3 target genes such as *VEGFA*, *IL6, TNFRSF1A*, and *TGFB1*, and impeding oncogenic processes such as angiogenesis, VM, tumor cell motility, and metastasis (Fig. 8F). Collectively, our results demonstrate that αEGFR-E-P125A downregulates signaling through effects on both EGFR and α5β1 integrin, simultaneously suppressing two compensatory pathways.

While the induction of VM has been attributed to the activation of signaling pathways such as NOTCH, FAK, VEGF, WNT, and HIFα signaling (7), a gene signature corresponding to both the induction and inhibition of VM has not been characterized previously. We utilized RNA-seq on MDA-MB-231-4175 tumor cells plated in 2D, in 3D on Matrigel, and in 3D and treated with αEGFR-E-P125A to define a set of signature genes and pathways that were upregulated upon the induction of VM, and downregulated upon VM inhibition by αEGFR-E-P125A. Key pathways inhibited by αEGFR-E-P125A include the IFN alpha response, IL6-JAK-STAT3, IFN gamma response, and angiogenesis signaling pathways. In these studies, we focused on the IL6-JAK-STAT3 and angiogenesis signaling pathways and the changes associated with these pathways in both the transcriptome and phosphoproteome and related these changes to VM because of the predicted roles of cetuximab and endostatin independently. Cetuximab is an anti-EGFR antibody known to downregulate downstream EGFR signaling, including signaling through STAT3 (5). STAT3 is a transcription factor whose phosphorylation at Y705 is known to promote the progression of epithelial-to-mesenchymal transition and has therefore been previously linked to VM; however, its specific role in VM has not been extensively studied and is not well understood (10). Here, we showed that targeted inhibition of STAT3 phosphorylation by the JAK2 inhibitor ruxolitinib, or the STAT3-specific inhibitor Cryptotanshinone, or indirect inhibition of STAT3 phosphorylation by upstream inhibition of the α5β1 integrin/FAK signaling cascade by dimeric presentation of E-P125A prevents VM tube formation *in vitro*.

Although the angiogenesis signaling pathway was characterized for endothelial cells, it has also been identified as a mechanistically parallel process to VM formation (6–8). Because of the antiangiogenic effects of E-P125A (14), we investigated the effects of αEGFR-E-P125A on angiogenic signaling genes and proteins in tumor cells themselves and found decreased expression of angiogenic genes such as *VEGFA*, *PTK2*, *ANGPT1*, *ITGAV*, and *ITGA5*, and decreased phosphorylation of FAK protein at Y397 and total levels of α5 integrin. In addition to targeting α5β1 integrin, endostatin can bind to various angiogenic receptors such as VEGFR2, αvβ3 integrin, glypicans, nucleolin, and the Wnt receptors LRP5/6 and Frizzled (51). While our initial studies primarily emphasized αEGFR-E-P125A's effects on α5β1 integrin, in future studies we can further explore the effect of αEGFR-E-P125A treatment on additional endostatin binding targets. To draw a comparison between the mechanisms of VM and angiogenesis, we are currently analyzing the dysregulation of the angiogenic gene set upon treatment of HUVEC cells with αEGFR-E-P125A to identify genes that are essential for the process of tube formation in both cell types. A comparison of the mechanisms controlling VM in tumor cells and angiogenesis in endothelial cells is a subject for future investigation.

Two additional pathways which were identified as inversely regulated during VM induction and its inhibition by αEGFR-E-P125A were the IFN alpha and IFN gamma immune response pathways. The effects of cetuximab and E-P125A treatment on IFN gamma and alpha immune response pathways have not been well characterized. Therefore, identification of the two pathways from our RNA-seq data is a novel unexpected finding regarding the combined effects of cetuximab and E-P125A on tumor cells and the process of VM, and is a subject for future investigation.

The current clinical standard of care for TNBC treatment includes surgery, radiation, and neoadjuvant chemotherapy for primary tumors, whereas chemotherapy, PARP inhibitors, and immunotherapeutic anti-checkpoint agents are used for metastatic disease (52). However, current monotherapies have limited therapeutic efficacy, demonstrating the clinical need for more effective targeted therapies for the treatment of TNBC. The potential advantage of αEGFR-E-P125A over standard treatments is that the αEGFR-E-P125A antibody fusion protein can utilize a mechanism of dual inhibition to simultaneously downregulate both EGFR and α5β1 integrin signaling. Standard monotherapies such as cetuximab or voloxicimab can suppress signaling through either EGFR or α5β1 integrin respectively (5, 15, 53); however, because of cross-talk between their downstream signaling cascades, monotherapies frequently fail due to the activation of compensatory mechanisms and confer resistance to therapy in TNBC (23, 24, 27, 54). Studies on integrins in breast cancer and pancreatic ductal adenocarcinoma have demonstrated that integrin signaling can increase the secretion of EGF, which may bypass the blocking effect of mAbs, such as cetuximab. Alternate pathways which can compensate for EGFR inhibition and promote drug resistance include RAS-MAPK, PI3K-Akt, STAT, and Src signaling, though they were not all explored in this study (55). Conversely, the inhibition of integrin signaling has been shown to reverse this therapeutic resistance and sensitize cells to cetuximab (47). Unlike standard monotherapies, the αEGFR-E-P125A fusion protein inhibits both EGFR and α5β1 integrin simultaneously, preventing the compensatory induction of signaling.

αEGFR-E-P125A inhibits α5β1 integrin/FAK signaling, which affects motility, metastasis, and tumor aggressiveness. αEGFR-E-P125A was shown to inhibit primary tumor growth, but we also observed a notable reduction in metastasis *in vivo* in our previous work (12), as well as reduced lung metastasis in this study following orthotopic implantation. As the 5-year survival rate of patients with TNBC is significantly lower than that of other breast cancers due to increased metastasis, utilizing a therapy such as αEGFR-E-P125A, which inhibits α5β1 integrin/FAK-mediated motility and tumor cell invasiveness is a promising therapeutic approach for patients with TNBC (56).

In this study, we demonstrated the benefits of using the αEGFR-E-P125A antibody fusion protein as a monotherapy with dual inhibitory functionality. To further enhance the inhibitory effects of αEGFR-E-P125A, it would be clinically relevant to also study the effects of αEGFR-E-P125A in combination with chemotherapy, PARP inhibitors, and/or checkpoint inhibitors currently used in TNBC. The IL6-JAK-STAT3 signaling pathway is known to promote tumor cell resistance to chemotherapy (40) and αEGFR-E-P125A treatment downregulates IL6-JAK-STAT3 signaling. The combination of αEGFR-E-P125A with chemotherapy to reduce chemoresistance is currently under investigation.

As a therapy designed to target EGFR-overexpressing tumors, αEGFR-E-P125A has the potential to target a broad range of additional EGFR-overexpressing cancers with high metastatic propensity. Because of its combined inhibition of EGFR and α5β1 integrin/FAK signaling, as well as its downregulation of the IL6-JAK-STAT3 and angiogenesis signaling pathways, αEGFR-E-P125A treatment has the potential to overcome both the compensatory signaling associated with monotherapies and the chemoresistance typically associated with TNBC, thereby reducing TNBC metastasis.

## Supplementary Material

Supplementary Figure 1A Extended heatmap of differentially expressed genes

Supplementary Figure 1B Extended heatmap of differentially expressed genes

Supplementary Figure 2 Extended heatmap of differentially expressed genes

Supplementary Figure 3 GSEA of Hallmarks pathways downregulated from 2D to 3D and upregulated upon αEGFR-E-P125A treatment

Supplementary Figure 4 αEGFR-E-P125A treatment timecourse

Supplementary Figure 5 Dimeric form of E-P125A (FcE) demonstrates enhanced inhibition of VM tube formation

Supplementary Figure 6 Effects of α5β1 integrin and its downstream signaling components on VM tube formation

Supplementary Figure 7 Effects of Cryptotanshinone (CPT), a STAT3 Y705-specific inhibitor on VM tube formation

Supplementary Figure 8 Expression levels of genes of interest across human breast cancer cell lines

Supplementary Figure 9 Expression levels of genes of interest across human breast cancer subtypes

Supplementary Table 1-1 Extended table of differentially expressed genes from 2D to 3D

Supplementary Table 1-2 Extended table of differentially expressed genes from 2D to 3D

Supplementary Table 1-3 Extended table of differentially expressed genes from 2D to 3D

Supplementary Table 1-4 Extended table of differentially expressed genes from 2D to 3D

Supplementary Table 2 Extended table of differentially expressed genes from 3D to αEGFR-E-P125A
