# Supplementary material for: Antibody–Drug Conjugate αEGFR-E-P125A Reduces Triple-negative Breast Cancer Vasculogenic Mimicry, Motility, and Metastasis through Inhibition of EGFR, Integrin, and FAK/STAT3 Signaling
Source: Cancer Res Commun. 2024 Mar 11;4(3):738–56. doi: 10.1158/2767-9764.CRC-23-0278 (PMC10926898; doi:10.1158/2767-9764.CRC-23-0278)
Supplement: Supplementary Figure 1A — Extended heatmap of differentially expressed genes [file crc-23-0278-s01.pdf]

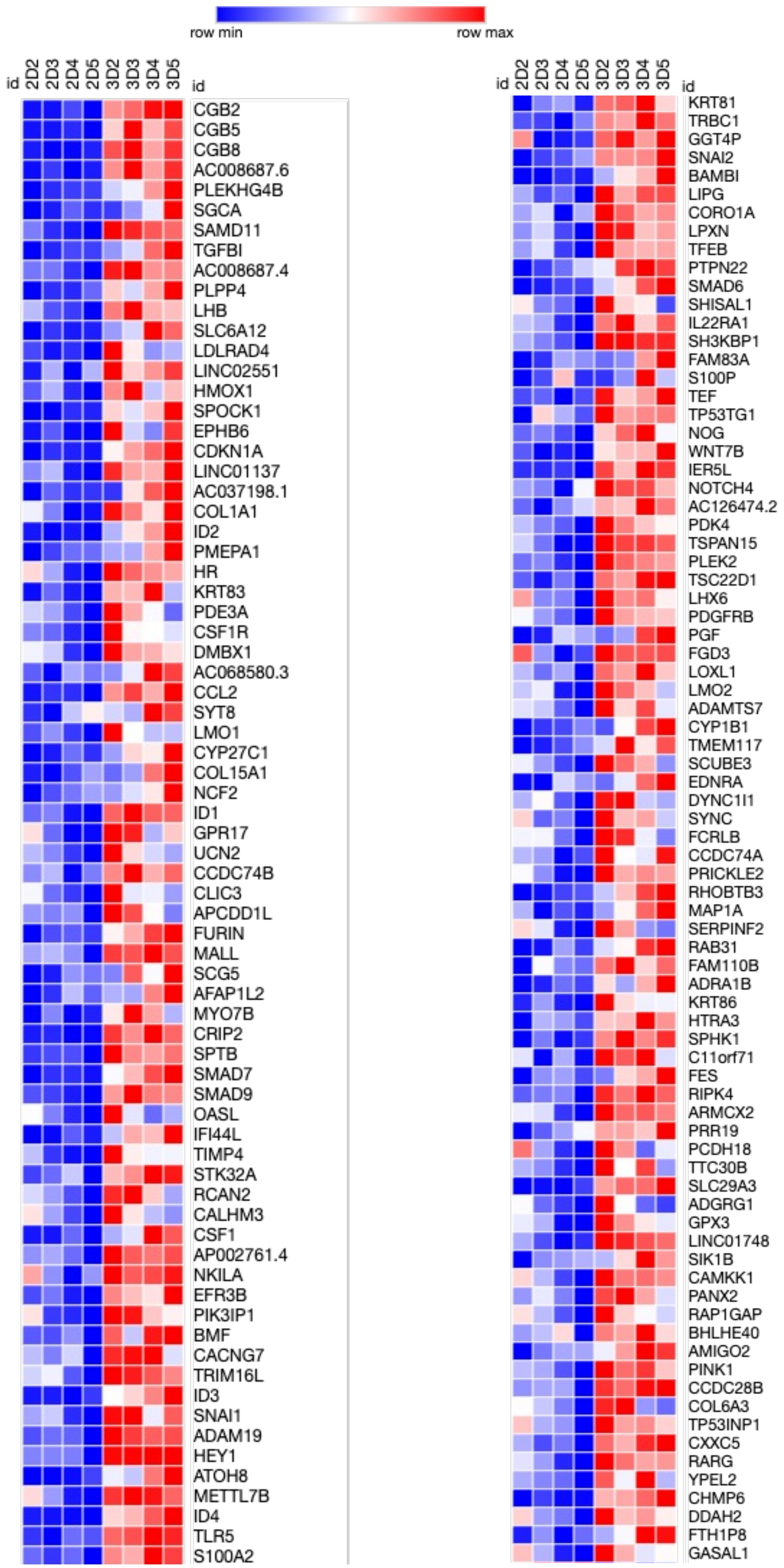

Supplementary Figure 1. Extended heatmap of differentially expressed genes. Heatmap lists differentially expressed genes upregulated from the 2D to 3D treatment transition. Heatmap was generated using the Broad Institute’s Morpheus analysis software.
