# Supplementary material for: Antibody–Drug Conjugate αEGFR-E-P125A Reduces Triple-negative Breast Cancer Vasculogenic Mimicry, Motility, and Metastasis through Inhibition of EGFR, Integrin, and FAK/STAT3 Signaling
Source: Cancer Res Commun. 2024 Mar 11;4(3):738–56. doi: 10.1158/2767-9764.CRC-23-0278 (PMC10926898; doi:10.1158/2767-9764.CRC-23-0278)
Supplement: Supplementary Figure 2 — Extended heatmap of differentially expressed genes [file crc-23-0278-s03.pdf]

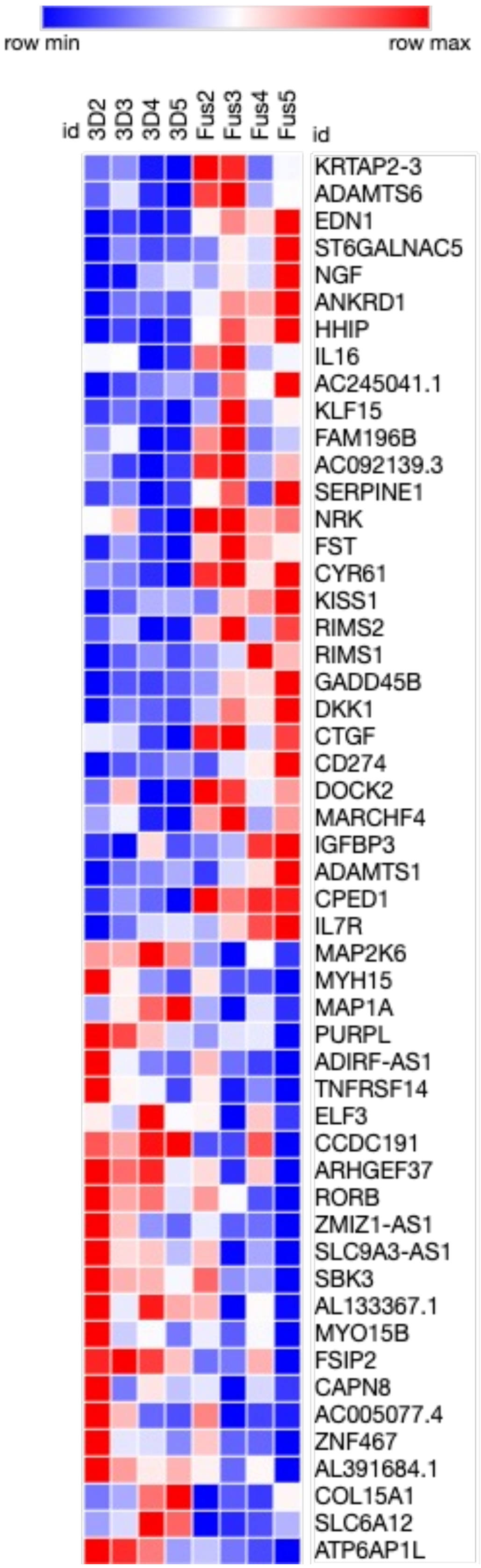

Supplementary Figure 2. Extended heatmap of differentially expressed genes. Heatmap lists differentially expressed genes dysregulated from the 3D to αEGFR-E-P125A treatment transition. Heatmap was generated using the Broad Institute's Morpheus analysis software.
