# Supplementary figures and images for: Antibody–Drug Conjugate αEGFR-E-P125A Reduces Triple-negative Breast Cancer Vasculogenic Mimicry, Motility, and Metastasis through Inhibition of EGFR, Integrin, and FAK/STAT3 Signaling
Source: Cancer Res Commun. 2024 Mar 11;4(3):738–56. doi: 10.1158/2767-9764.CRC-23-0278 (PMC10926898; doi:10.1158/2767-9764.CRC-23-0278)

## G2M Checkpoint

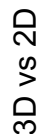

### αEGFR-E-P125A vs 3D

### Top Upregulated Pathways

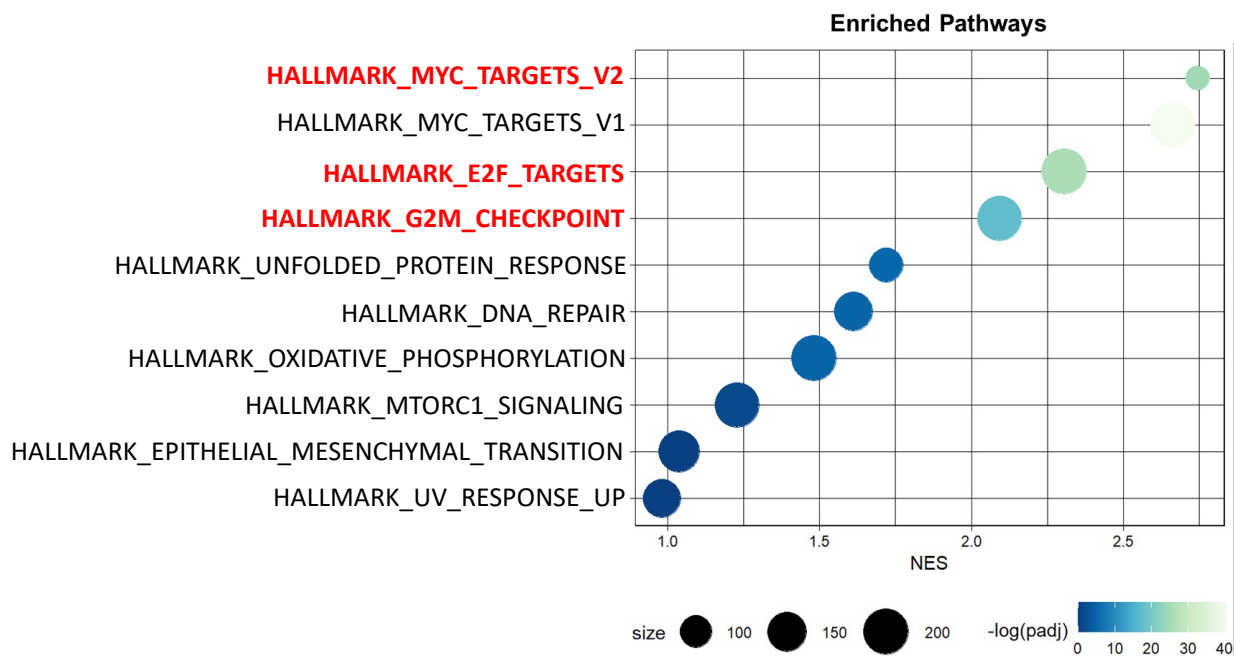

4

Supplement: Supplementary Figure 3 — GSEA of Hallmarks pathways downregulated from 2D to 3D and upregulated upon αEGFR-E-P125A treatment [file crc-23-0278-s04.pdf]
