# Supplementary material for: Antibody–Drug Conjugate αEGFR-E-P125A Reduces Triple-negative Breast Cancer Vasculogenic Mimicry, Motility, and Metastasis through Inhibition of EGFR, Integrin, and FAK/STAT3 Signaling
Source: Cancer Res Commun. 2024 Mar 11;4(3):738–56. doi: 10.1158/2767-9764.CRC-23-0278 (PMC10926898; doi:10.1158/2767-9764.CRC-23-0278)
Supplement: Supplementary Figure 4 — αEGFR-E-P125A treatment timecourse [file crc-23-0278-s05.pdf]

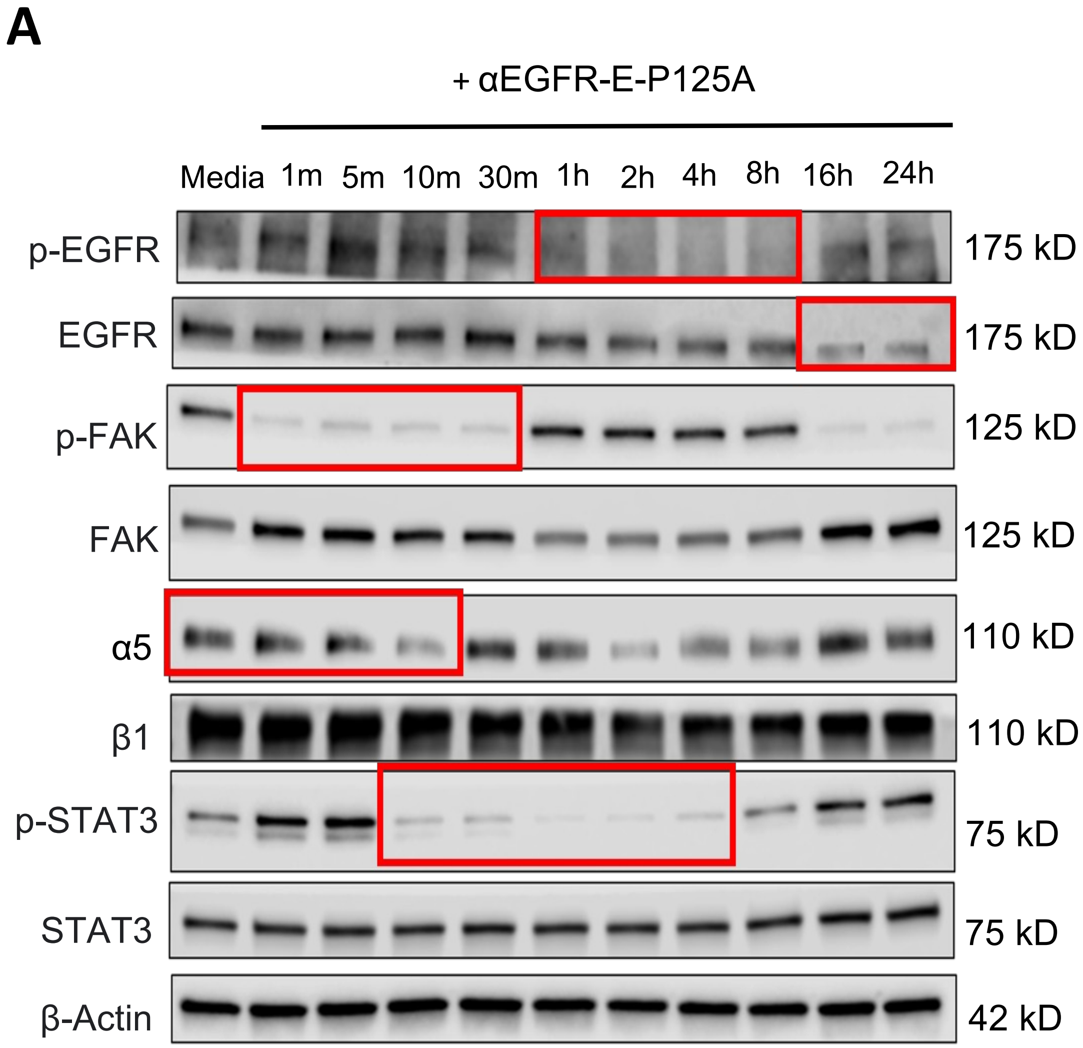

Supplementary Figure 4. αEGFR-E-P125A treatment timecourse**. A,** Western blot depicting signaling changes associated with both early and late time points upon αEGFR-E-P125A treatment of MDA-MB-231-4175 cells in 2D.
