# Supplementary material for: Antibody–Drug Conjugate αEGFR-E-P125A Reduces Triple-negative Breast Cancer Vasculogenic Mimicry, Motility, and Metastasis through Inhibition of EGFR, Integrin, and FAK/STAT3 Signaling
Source: Cancer Res Commun. 2024 Mar 11;4(3):738–56. doi: 10.1158/2767-9764.CRC-23-0278 (PMC10926898; doi:10.1158/2767-9764.CRC-23-0278)
Supplement: Supplementary Figure 5 — Dimeric form of E-P125A (FcE) demonstrates enhanced inhibition of VM tube formation [file crc-23-0278-s06.pdf]

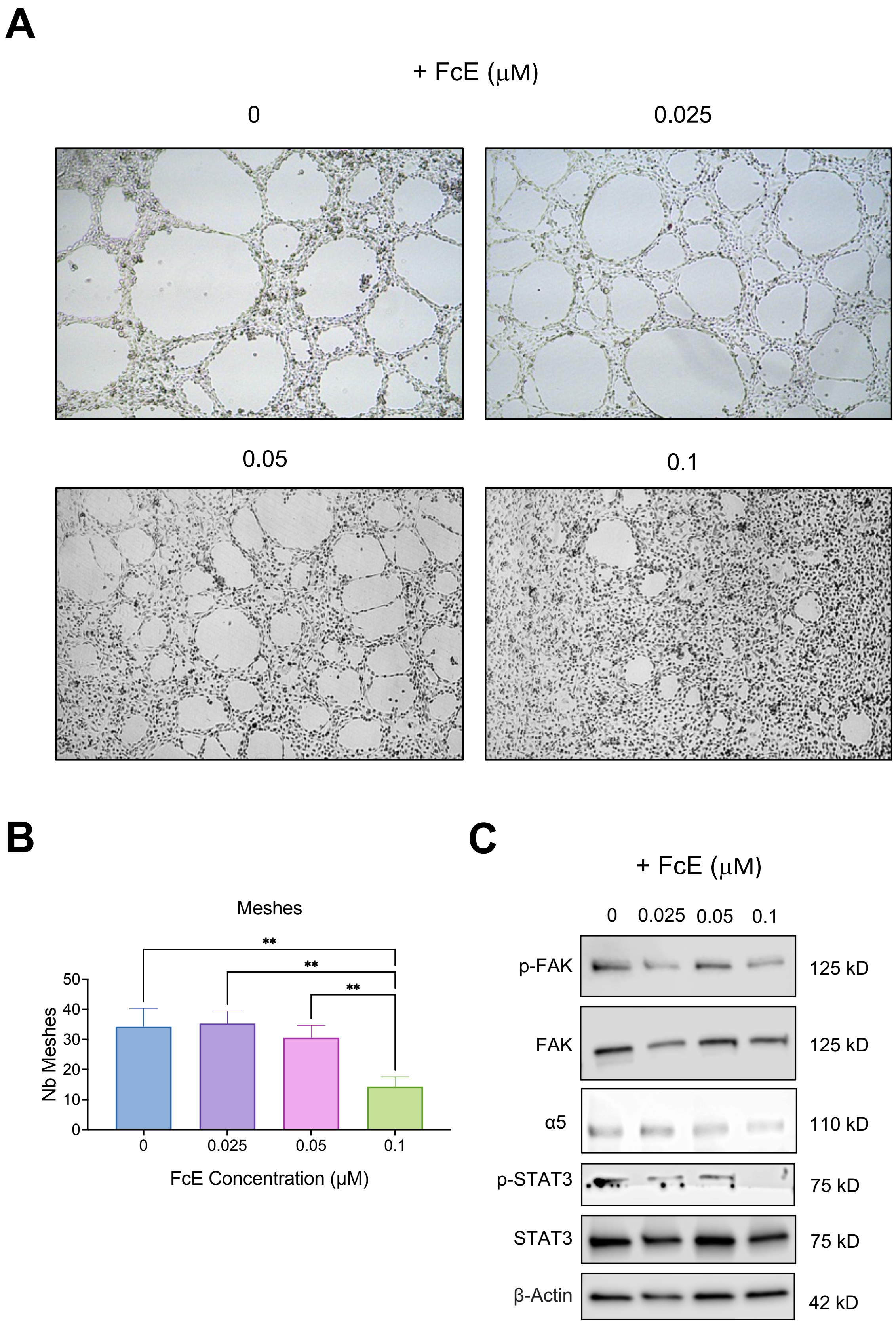

Supplementary Figure 5. Dimeric form of E-P125A (FcE), demonstrates enhanced inhibition of VM tube formation**. A,** Dimeric form of E-P125A (FcE) enhances the inhibitory effects of VM tube formation with with increasing doses. **B,** Increasing doses of FcE decreases the number of VM meshes formed. **C,** Western blot demonstrates that FcE treated TNBC cells have decreased p-FAK, p-STAT3, and α5 integrin protein levels in a dose dependent manner. **P < 0.01
