# Supplementary material for: Antibody–Drug Conjugate αEGFR-E-P125A Reduces Triple-negative Breast Cancer Vasculogenic Mimicry, Motility, and Metastasis through Inhibition of EGFR, Integrin, and FAK/STAT3 Signaling
Source: Cancer Res Commun. 2024 Mar 11;4(3):738–56. doi: 10.1158/2767-9764.CRC-23-0278 (PMC10926898; doi:10.1158/2767-9764.CRC-23-0278)
Supplement: Supplementary Figure 6 — Effects of α5β1 integrin and its downstream signaling components on VM tube formation [file crc-23-0278-s07.pdf]

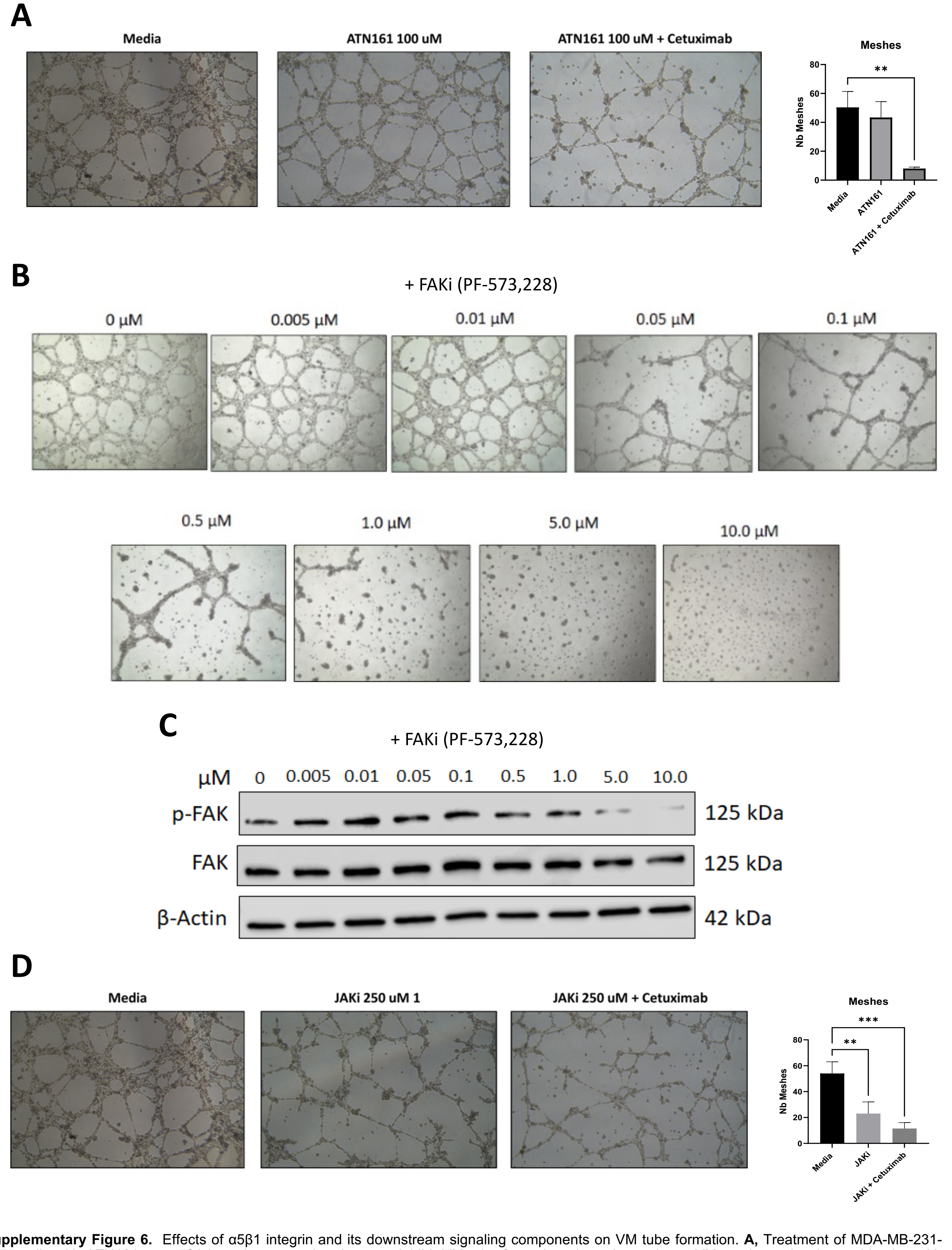

Supplementary Figure 6. Effects of α5β1 integrin and its downstream signaling components on VM tube formation. **A,** Treatment of MDA-MB-231-4175 cells with ATN161, an α5β1 integrin antagonist, does not inhibit VM tube formation alone, but reduces VM mesh number in combination with Cetuximab. **B,** FAK inhibitor, PF-573228 inhibits VM tube formation in a dose dependent manner in MDA-MB-231-4175 cells plated on matrigel. **C,** Western blot representing downregulation of P-FAK upon treatment with PF-573228 in a dose-dependent manner. **D,** Treatment of MDA-MB-231-4175 cells with Ruxolitinib, a JAK inhibitor alone reduces, but does not completely inhibit VM tube formation, but when treated in combination with Cetuximab, there is an enhanced inhibition of VM mesh number as well as reduction of STAT3 phosphorylation at the Y705 site.
