# Supplementary material for: Antibody–Drug Conjugate αEGFR-E-P125A Reduces Triple-negative Breast Cancer Vasculogenic Mimicry, Motility, and Metastasis through Inhibition of EGFR, Integrin, and FAK/STAT3 Signaling
Source: Cancer Res Commun. 2024 Mar 11;4(3):738–56. doi: 10.1158/2767-9764.CRC-23-0278 (PMC10926898; doi:10.1158/2767-9764.CRC-23-0278)
Supplement: Supplementary Figure 7 — Effects of Cryptotanshinone (CPT), a STAT3 Y705-specific inhibitor on VM tube formation [file crc-23-0278-s08.pdf]

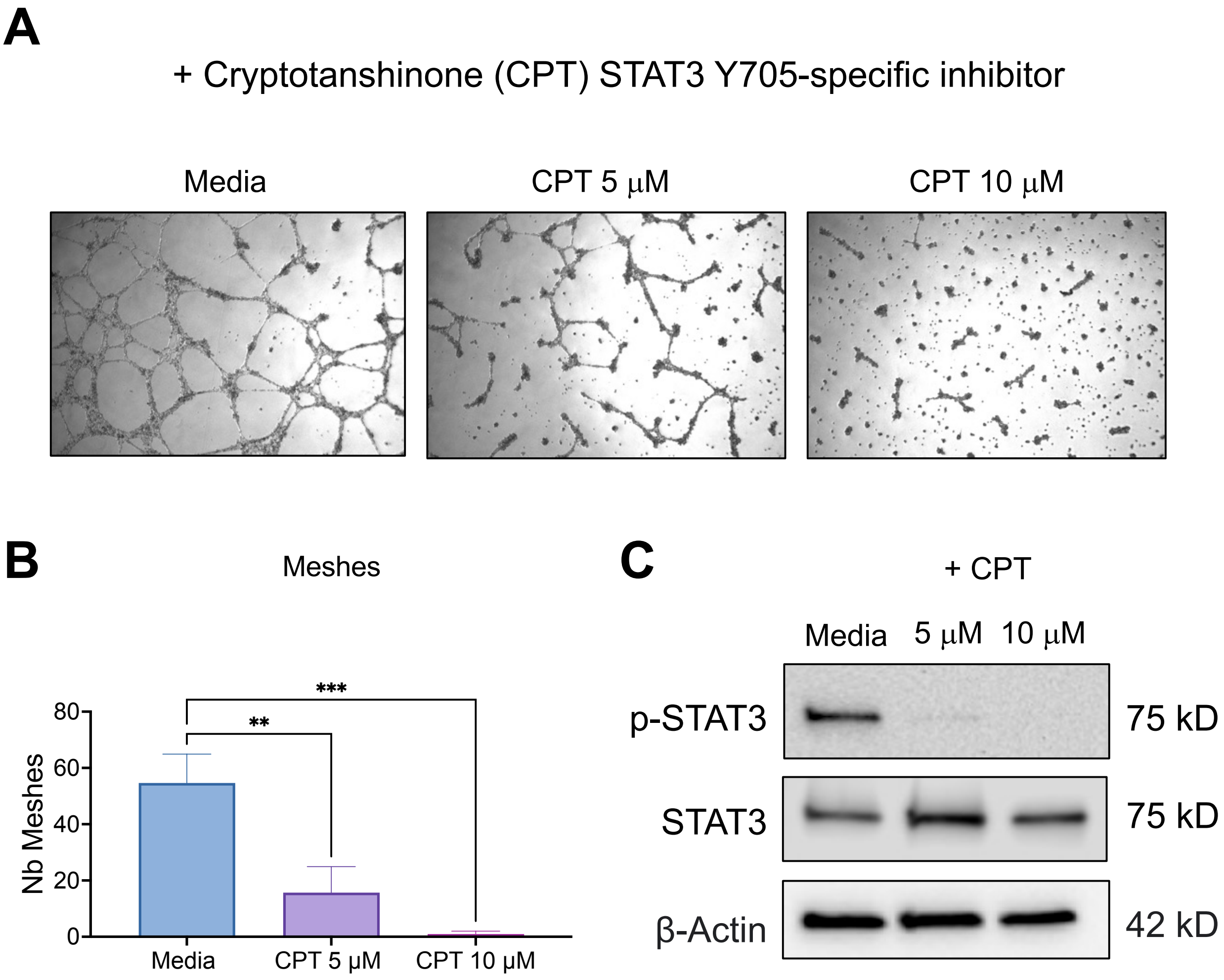


**Supplementary Figure 7.** Effects of Cryptotanshinone (CPT), a STAT3 Y705-specific inhibitor on VM tube formation. **A,** Dose-dependent response of CPT on VM tube formation demonstrates inhibition of VM tube formation with increasing CPT doses. **B**, Quantification of VM tube formation of CPT-treated cells depicts reduced mesh number with increasing doses of CPT. **C**, Western blot analysis from samples of cells treated with increasing doses of CPT verifies inhibition of p-STAT3 at the Y705 phosphorylation site.
