# Supplementary material for: Antibody–Drug Conjugate αEGFR-E-P125A Reduces Triple-negative Breast Cancer Vasculogenic Mimicry, Motility, and Metastasis through Inhibition of EGFR, Integrin, and FAK/STAT3 Signaling
Source: Cancer Res Commun. 2024 Mar 11;4(3):738–56. doi: 10.1158/2767-9764.CRC-23-0278 (PMC10926898; doi:10.1158/2767-9764.CRC-23-0278)
Supplement: Supplementary Figure 8 — Expression levels of genes of interest across human breast cancer cell lines [file crc-23-0278-s09.pdf]

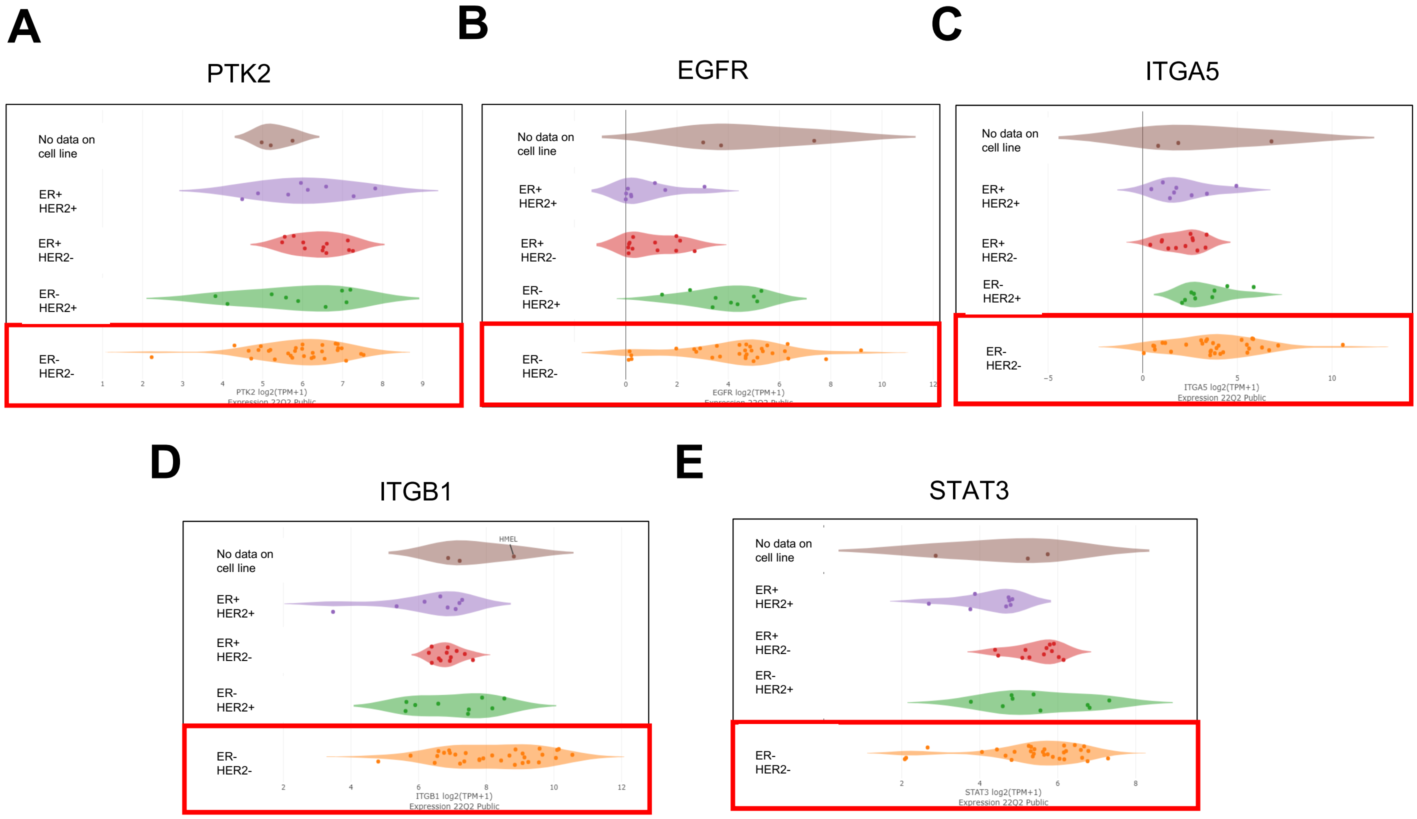

Supplementary Figure 8. Expression levels of genes of interest across human breast cancer cell lines. **A-E,** Cancer Dependency Map (DepMap 2023) portal plots demonstrating higher FAK, EGFR, ITGA5, ITGB1, and STAT3 expression in TNBC subtypes, respectively.
