# Supplementary material for: Antibody–Drug Conjugate αEGFR-E-P125A Reduces Triple-negative Breast Cancer Vasculogenic Mimicry, Motility, and Metastasis through Inhibition of EGFR, Integrin, and FAK/STAT3 Signaling
Source: Cancer Res Commun. 2024 Mar 11;4(3):738–56. doi: 10.1158/2767-9764.CRC-23-0278 (PMC10926898; doi:10.1158/2767-9764.CRC-23-0278)
Supplement: Supplementary Table 1-1 — Extended table of differentially expressed genes from 2D to 3D [file crc-23-0278-s11.pdf]

Gene_name	log2FoldChange	padj	Direction
CGB2	2.99	5.71E-08	up
CGB5	2.88	7.38E-07	up
CGB8	2.38	0	up
AC008687.6	2.38	0	up
PLEKHG4B	2.34	0	up
SGCA	2.2	1.76E-05	up
SAMD11	1.79	0	up
AC008687.4	1.55	0	up
TGFBI	1.52	0	up
PLPP4	1.5	6.82E-08	up
LHB	1.45	2.49E-06	up
SLC6A12	1.39	0.000487	up
LDLRAD4	1.39	0.00742	up
LINC02551	1.37	0.0011	up
HMOX1	1.35	1.62E-08	up
SPOCK1	1.31	0	up
EPHB6	1.28	0.0146	up
AC037198.1	1.26	3.12E-06	up
CDKN1A	1.24	0	up
LINC01137	1.24	0.000157	up
HR	1.22	9.88E-05	up
COL1A1	1.21	3.83E-06	up
ID2	1.21	0	up
DMBX1	1.21	0.00164	up
AC068580.3	1.21	0.00122	up
PMEPA1	1.2	8.08E-07	up
KRT83	1.19	0.00132	up
CSF1R	1.19	6.03E-07	up
PDE3A	1.17	0.00451	up
COL15A1	1.15	0.002	up
LMO1	1.14	0.0175	up
CCL2	1.12	0	up
SYT8	1.12	0.00656	up
CYP27C1	1.12	9.89E-05	up
ID1	1.1	0	up
UCN2	1.1	0	up
APCDD1L	1.1	0.00221	up
CLIC3	1.09	6.6E-09	up
NCF2	1.07	4.75E-08	up
GPR17	1.06	6.74E-05	up
CCDC74B	1.05	0.0151	up
MYO7B	1.03	8.01E-05	up
AFAP1L2	1.02	8.11E-08	up
CRIP2	1.02	6E-10	up
MALL	0.99	5.31E-08	up
SCG5	0.973	2.48E-05	up
FURIN	0.97	0	up
SMAD7	0.962	0	up
CALHM3	0.949	0.000114	up
IFI44L	0.942	8.36E-06	up
OASL	0.937	1.78E-07	up
TIMP4	0.932	0	up
NKILA	0.917	0.0262	up
SPTB	0.914	0	up
AP002761.4	0.913	0.000482	up
SMAD9	0.905	0	up
STK32A	0.9	1.8E-09	up
RCAN2	0.894	0.000175	up
CSF1	0.889	0	up
ADAM19	0.887	0	up
HEY1	0.871	0.000672	up
BMF	0.867	0.000028	up
TLR5	0.854	0.00417	up
GGT4P	0.842	0.0482	up
METTL7B	0.838	9.9E-07	up
TRIM16L	0.834	0	up
ID3	0.833	0	up
PIK3IP1	0.827	0.00208	up
KRT81	0.824	0	up
SNAI1	0.822	3.22E-07	up
ID4	0.819	0	up
ATOH8	0.816	0	up
S100A2	0.816	0	up
TRBC1	0.814	0.0122	up
EFR3B	0.812	4.58E-08	up
CACNG7	0.808	0.0013	up
IL22RA1	0.807	0.0041	up
FAM83A	0.801	0.0000681	up
IER5L	0.8	0	up
TFEB	0.799	0.000004	up
LPXN	0.798	0	up
PTPN22	0.792	0.00056	up
SMAD6	0.792	0	up
BAMBI	0.784	0	up
WNT7B	0.773	1.6E-09	up
SH3KBP1	0.769	0	up
SNAI2	0.768	0	up
S100P	0.759	0.000983	up
AC126474.2	0.758	0.00123	up
TP53TG1	0.755	0.0259	up
NOTCH4	0.741	0.0277	up
LIPG	0.736	0	up
CORO1A	0.735	0.016	up
TEF	0.733	0	up
NOG	0.731	0	up
SHISAL1	0.728	1E-10	up
FGD3	0.72	0.00324	up
LOXL1	0.713	0	up
LHX6	0.712	0.00178	up
LMO2	0.712	0.0051	up
SERPINF2	0.709	0.0096	up
FES	0.705	0.0345	up
TSC22D1	0.704	0	up
PRICKLE2	0.704	0	up
DYNC1I1	0.698	0.000598	up
CCDC74A	0.698	0.00331	up
HTRA3	0.696	0	up
ADGRG1	0.696	0	up
FCRLB	0.694	0.00657	up
LINC01748	0.69	0.0404	up
RIPK4	0.685	0	up
CYP1B1	0.684	0.000000467	up
MAP1A	0.681	0.0064	up
PRR19	0.679	0.014	up
PDK4	0.673	0.00000101	up
PCDH18	0.673	0.0439	up
PDGFRB	0.672	0	up
SLC29A3	0.672	0	up
SYNC	0.668	0.00125	up
TSPAN15	0.666	0	up
SPHK1	0.664	0	up
C11orf71	0.664	0.0411	up
PGF	0.662	0	up
EDNRA	0.661	1.38E-08	up
KRT86	0.66	0.0104	up
TMEM117	0.655	0.0132	up
ARMCX2	0.655	0.0000137	up
RAB31	0.654	0	up

Supplementary Table 1. Extended table of differentially expressed genes from 2D to 3D. Table of differentially expressed genes upregulated from the 2D to 3D treatment transition. Table lists gene name, log2fc, padjusted value (padj), and direction of dysregulation from 2D to 3D.
