# Supplementary material for: Antibody–Drug Conjugate αEGFR-E-P125A Reduces Triple-negative Breast Cancer Vasculogenic Mimicry, Motility, and Metastasis through Inhibition of EGFR, Integrin, and FAK/STAT3 Signaling
Source: Cancer Res Commun. 2024 Mar 11;4(3):738–56. doi: 10.1158/2767-9764.CRC-23-0278 (PMC10926898; doi:10.1158/2767-9764.CRC-23-0278)
Supplement: Supplementary Table 1-2 — Extended table of differentially expressed genes from 2D to 3D [file crc-23-0278-s12.pdf]

Gene_name	log2FoldChange	padj	Direction
ADRA1B	0.654	0.000000586	up
TTC30B	0.654	0.0283	up
ADAMTS7	0.649	0.00157	up
SCUBE3	0.645	0.00000462	up
PLEK2	0.644	0	up
SIK1B	0.644	0.00463	up
RHOBTB3	0.64	0	up
GPX3	0.638	0	up
FAM110B	0.637	0.0469	up
CHMP6	0.63	0	up
CXXC5	0.629	0	up
DDAH2	0.629	0	up
RAP1GAP	0.624	0.000246	up
CCDC28B	0.622	0.0000791	up
PANX2	0.619	0	up
TP53INP1	0.618	0.00313	up
CAMKK1	0.616	2.64E-08	up
AMIGO2	0.615	0	up
COL6A3	0.615	0.00544	up
PINK1	0.614	0	up
RARG	0.613	0	up
FTH1P8	0.609	0.0139	up
BHLHE40	0.607	0.000000637	up
GASAL1	0.602	0.00711	up
YPEL2	0.601	0.00389	up

Supplementary Table 1 (Continued). Extended table of differentially expressed genes from 2D to 3D. Table of differentially expressed genes upregulated from the 2D to 3D treatment transition. Table lists gene name, log2fc, padjusted value (padj), and direction of dysregulation from 2D to 3D.
