# Supplementary material for: Antibody–Drug Conjugate αEGFR-E-P125A Reduces Triple-negative Breast Cancer Vasculogenic Mimicry, Motility, and Metastasis through Inhibition of EGFR, Integrin, and FAK/STAT3 Signaling
Source: Cancer Res Commun. 2024 Mar 11;4(3):738–56. doi: 10.1158/2767-9764.CRC-23-0278 (PMC10926898; doi:10.1158/2767-9764.CRC-23-0278)
Supplement: Supplementary Table 1-3 — Extended table of differentially expressed genes from 2D to 3D [file crc-23-0278-s13.pdf]

Gene_name	log2FoldChange	padj	Direction
DDX47	-0.6	0.00164	down
LINC01836	-0.6	0.0445	down
ZNF91	-0.601	0.00242	down
SLC25A25-AS1	-0.601	0.000513	down
SGO2	-0.602	0	down
NKTR	-0.603	2E-10	down
ENTPD4	-0.603	0	down
SNHG1	-0.604	0	down
SLC7A2	-0.606	0	down
GCSH	-0.606	0.0463	down
SLC27A3	-0.606	0.00339	down
DLEU2	-0.609	0.00013	down
SNAPC1	-0.613	0	down
SPACA6	-0.613	0.0068	down
FAT1	-0.614	0	down
F3	-0.615	0	down
AASS	-0.617	0	down
ZNF107	-0.618	6.06E-08	down
LINC00641	-0.618	1.9E-09	down
AC022400.7	-0.618	0.000378	down
ADAMTSL4	-0.619	0.011	down
MIR222HG	-0.619	0.0000702	down
SAMD4A	-0.621	0	down
MDN1	-0.621	0.000000005	down
AC138393.1	-0.621	0.0207	down
OR51B5	-0.623	0.0452	down
DKK1	-0.626	0	down
AL591845.1	-0.627	0.00113	down
SCNN1D	-0.627	0.000156	down
SRRM2	-0.628	0	down
MT-ND1	-0.628	0	down
TNFRSF11B	-0.629	0.0259	down
AC091057.5	-0.629	0.00905	down
ARHGAP20	-0.63	0.00297	down
NOTCH3	-0.631	0.00181	down
AC008105.3	-0.632	0.00165	down
SNHG4	-0.632	4E-10	down
EFEMP1	-0.634	7.47E-08	down
EFEMP2	-0.637	0.0265	down
CTGF	-0.638	0.0000528	down
FAM196B	-0.639	0.00000107	down
NAV2	-0.64	0	down
ZNF695	-0.64	0.0417	down
DDX12P	-0.641	0.0000101	down
UGCG	-0.643	0	down
SLAMF7	-0.645	1E-10	down
SYNE2	-0.645	0.0000545	down
AC005674.2	-0.646	0.0259	down
ZC3H12B	-0.647	0.0483	down
KCNQ3	-0.647	0	down
KCNQ1OT1	-0.647	0.0000027	down
AC092821.1	-0.65	0.023	down
CRIM1	-0.652	0	down
FLNB-AS1	-0.654	0.00433	down
PKP2	-0.656	0	down
MT-ND6	-0.659	7.8E-09	down
DAW1	-0.66	0.0441	down
AKAP9	-0.662	0	down
AC073335.2	-0.663	0.00112	down
HSPG2	-0.665	3.66E-08	down
AC104581.4	-0.666	0.0107	down
OTUD6B-AS1	-0.667	0	down
TGFB2	-0.668	0	down
ADRB2	-0.668	0	down
HERC2P2	-0.668	0	down
MND1	-0.67	0	down
BCL2A1	-0.67	0.0447	down
PRKG1-AS1	-0.671	0.0104	down
NEAT1	-0.672	0.000263	down
TIAF1	-0.674	0.00721	down
NSUN5P1	-0.675	0.00000177	down
LTB4R2	-0.676	0.000000144	down
SCN5A	-0.678	9.19E-08	down
FN1	-0.679	0	down
FER1L4	-0.681	0.03	down
GPRC5A	-0.682	2E-10	down
MNS1	-0.684	3.56E-08	down
INHBB	-0.684	0.000242	down
AC011498.7	-0.684	0.0000184	down
SEC31B	-0.685	1E-10	down
AL358113.1	-0.686	0.0225	down
SLFNL1-AS1	-0.688	0.0365	down
CENPK	-0.691	0	down
AC104162.2	-0.694	0.0341	down
TPRKB	-0.695	0	down
GPR135	-0.695	1.66E-08	down
AL136164.4	-0.696	0.00389	down
AC027237.3	-0.7	0.0306	down
KLRA1P	-0.706	0.00253	down
AC008560.1	-0.708	0.0126	down
SMPD3	-0.71	0.000733	down
MSH5	-0.71	0.000626	down
NR2F2-AS1	-0.711	0.0222	down
SGK494	-0.713	0.0000387	down
MT-ND5	-0.713	0.000000247	down
CAPN10-AS1	-0.715	0.00808	down
NEDD4L	-0.716	0	down
CCDC14	-0.716	0	down
AC000123.3	-0.717	0.000608	down
AC103718.1	-0.718	0.0331	down
PRR7-AS1	-0.72	0.0408	down
CCDC150	-0.723	0.000000552	down
RAB26	-0.723	0.0109	down
C20orf204	-0.723	0.0266	down
CENPQ	-0.724	0	down
LINC00632	-0.727	0.0134	down
ANKRD18A	-0.728	0.000000343	down
NTM	-0.728	0	down
ANKDD1A	-0.73	0.000736	down
AC241952.1	-0.73	0.0216	down
LENG8	-0.732	2.2E-09	down
DNAH11	-0.733	0.0000361	down
CSF2RA	-0.733	0.000012	down
MROH6	-0.733	0.00000013	down
NEDD9	-0.734	0.000132	down
DST	-0.734	0	down
LAMA5	-0.737	0	down
AC107375.1	-0.737	0.0292	down
SCML2	-0.74	0	down
C8orf59	-0.741	0	down
AC009022.1	-0.741	0.000307	down
AC008741.2	-0.741	0.00839	down
NR2F2	-0.742	0	down
AMH	-0.746	0	down
MARCH1	-0.746	0.000205	down
CEP290	-0.748	0	down
AC087741.1	-0.748	0.00628	down
SNHG12	-0.75	0	down
AC138028.6	-0.75	0.00816	down
NFATC2	-0.752	0.000000443	down
GRPR	-0.752	0.00985	down
HHIP	-0.752	0.00149	down
CDK5RAP3	-0.753	0	down
SPTBN5	-0.753	0.0000595	down
AJUBA	-0.754	0	down
DENND2A	-0.757	0.00269	down
MALAT1	-0.764	0.00000435	down
AMOTL2	-0.769	0	down
AL137127.1	-0.769	0.0311	down
AL121845.3	-0.77	0.019	down
REC8	-0.771	0.0066	down
AC027020.2	-0.771	0.00659	down
CCBE1	-0.772	0	down
WDR90	-0.774	0	down
HAUS7	-0.777	0.000502	down
GOLGA8B	-0.777	2.04E-08	down
SNX18P3	-0.777	0.0119	down
FAM95C	-0.78	0.00166	down
LTB4R	-0.781	0	down
EME2	-0.785	2E-10	down

Supplementary Table 1 (Continued). Extended table of differentially expressed genes from 2D to 3D. Table of differentially expressed genes upregulated from the 2D to 3D treatment transition. Table lists gene name, log2fc, padjusted value (padj), and direction of dysregulation from 2D to 3D.
