# Supplementary material for: Antibody–Drug Conjugate αEGFR-E-P125A Reduces Triple-negative Breast Cancer Vasculogenic Mimicry, Motility, and Metastasis through Inhibition of EGFR, Integrin, and FAK/STAT3 Signaling
Source: Cancer Res Commun. 2024 Mar 11;4(3):738–56. doi: 10.1158/2767-9764.CRC-23-0278 (PMC10926898; doi:10.1158/2767-9764.CRC-23-0278)
Supplement: Supplementary Table 1-4 — Extended table of differentially expressed genes from 2D to 3D [file crc-23-0278-s14.pdf]

Gene_name	log2FoldChange	padj	Direction
GAL3ST3	-0.786	0.00808	down
ANKRD20A5P	-0.789	0.000139	down
COL8A1	-0.794	0	down
CD274	-0.795	0	down
AC009404.1	-0.797	0.0169	down
LHX4	-0.802	0.000316	down
PRICKLE1	-0.803	5E-10	down
ALMS1-IT1	-0.804	0.0342	down
ADGRG6	-0.809	0	down
AL354836.1	-0.809	0.0000671	down
PNN	-0.81	0	down
CPED1	-0.817	0	down
NFYC-AS1	-0.817	0.00288	down
ALPP	-0.819	8.4E-09	down
CHKB-CPT1B	-0.822	0.0385	down
AC026740.1	-0.824	0.0283	down
AC084125.2	-0.826	0.0498	down
HSF4	-0.829	2E-10	down
AC008735.2	-0.835	0.0199	down
IGFBP3	-0.836	0.00000225	down
AC132872.1	-0.838	0.00351	down
TERT	-0.839	0.000000193	down
PSMD6-AS2	-0.842	0.00786	down
SHANK2	-0.844	0.000000278	down
ZNF92	-0.845	0	down
ROBO2	-0.85	0.000127	down
ARHGAP33	-0.855	1.7E-09	down
AC112907.3	-0.855	0.00451	down
AC090826.1	-0.857	0.0101	down
EVA1B	-0.859	0.0133	down
FBXL8	-0.86	3E-10	down
AC010735.1	-0.865	0.00000256	down
IZUMO4	-0.872	0.0403	down
UCKL1-AS1	-0.881	0.000774	down
CREB5	-0.883	0	down
NEXN	-0.883	0	down
ANKRD36C	-0.887	0	down
GNB3	-0.892	0.000237	down
FOXN3-AS1	-0.893	0.046	down
ARL6IP4	-0.895	0.0219	down
IL12A	-0.896	0.00343	down
AC097059.2	-0.897	0.0392	down
AC020978.9	-0.9	0.000293	down
SFTA1P	-0.903	0	down
ANXA3	-0.904	0	down
AP002807.1	-0.907	0.00417	down
SGPP2	-0.913	0.0143	down
COL12A1	-0.915	0	down
TNFRSF13C	-0.915	0.0469	down
LPAR1	-0.916	0	down
CD274	-0.917	0	down
CENPE	-0.923	0	down
RHBDL1	-0.929	0.000000326	down
LY6G5B	-0.929	1.53E-08	down
RIMS2	-0.933	0	down
EVI2A	-0.935	0	down
AL136452.1	-0.936	0.0234	down
ADAMTS1	-0.937	0	down
NEGR1	-0.943	0	down
MIR17HG	-0.943	0.0000766	down
CEMIP	-0.947	0.0000015	down
AC073957.3	-0.949	0.00000195	down
SPNS2	-0.954	0.000852	down
MAMDC4	-0.96	0	down
AC103691.1	-0.961	0.0354	down
AC132872.3	-0.963	0.0289	down
MIR137HG	-0.964	0.000000157	down
VAC14-AS1	-0.966	0.0331	down
ESF1	-0.973	0	down
AC006128.1	-0.976	0.015	down
AC027796.4	-0.982	0.0385	down
CCDC78	-0.984	0.000000152	down
SH2D5	-0.987	0	down
LINC00342	-0.987	0.000235	down
FAM71D	-0.989	0.000345	down

Gene_name	log2FoldChange	padj	Direction
STXBP6	-0.993	0	down
AC016738.1	-0.995	0.0326	down
KISS1	-1	0	down
PRSS53	-1.01	0.000333	down
CHRNB1	-1.01	0	down
AL353763.2	-1.01	0.00000606	down
ATP6V0A4	-1.02	0.00278	down
AC004264.1	-1.02	0.0134	down
RTEL1-TNFRSF6B	-1.03	2E-10	down
PFDN4	-1.03	0	down
LOXL4	-1.03	0.00113	down
AP006623.1	-1.03	0.000227	down
GOLGA8A	-1.05	0.00625	down
EDN1	-1.06	0	down
PEAR1	-1.06	0	down
CAVIN2	-1.07	0	down
STAM-AS1	-1.07	0.00695	down
LRRC69	-1.08	0.00789	down
AC073130.1	-1.08	0	down
STK31	-1.09	0	down
RGMB-AS1	-1.09	0.000232	down
GAS6-AS2	-1.09	0.0236	down
YJEFN3	-1.1	0	down
IL16	-1.11	0.0013	down
DMBT1	-1.11	0.00986	down
KCNAB3	-1.12	0.000774	down
CAPN8	-1.12	0.00000447	down
NRK	-1.13	0.000000111	down
AC126323.1	-1.13	0	down
ATP8B1	-1.14	2.33E-08	down
RELN	-1.14	0.00858	down
IGFN1	-1.15	0	down
CLDN1	-1.16	0	down
AC040162.3	-1.16	0.00298	down
ADAMTS6	-1.17	0.00000081	down
CCDC148	-1.17	0.00000158	down
SCG2	-1.17	0.0152	down
ITGB2-AS1	-1.17	0.0124	down
SEMA3E	-1.18	4E-10	down
SNCAIP	-1.19	0.0225	down
RIMS1	-1.19	8.8E-09	down
IL7R	-1.19	0	down
AC108463.3	-1.19	0.018	down
FST	-1.2	0	down
ASMTL-AS1	-1.2	0.0456	down
SLC23A3	-1.22	0.00598	down
FGF14	-1.25	0.000132	down
AL390726.6	-1.25	0.00292	down
IL11	-1.26	0	down
GOLGA2P11	-1.26	0.000000061	down
TMEM236	-1.27	0.00114	down
NGF	-1.28	0.000798	down
SYNE4	-1.28	0.00203	down
MIR503HG	-1.3	2.73E-08	down
RPL13AP20	-1.3	0	down
AC144450.1	-1.32	0.00798	down
PGM5P2	-1.33	0	down
ANKRD1	-1.35	0	down
SRPK3	-1.37	0.0114	down
RGS7	-1.4	0.00115	down
DIO2	-1.47	0.00423	down
KLF15	-1.49	0.00000556	down
SCEL	-1.56	0.0000211	down
TPO	-1.57	0.000608	down
ST6GALNAC5	-1.59	0	down
MUC5B	-1.59	0	down
APLN	-1.61	3.2E-09	down
KRTAP2-3	-1.65	9E-10	down
CPA4	-1.68	0.000171	down
LINC00452	-1.71	0.0000121	down
PLCE1-AS1	-1.79	0.00000412	down
MUC5AC	-1.89	5.6E-09	down
PDE1C	-2.06	0.0000402	down
PTPRQ	-2.42	0.00000944	down
ESM1	-2.44	0.00000219	down
ANO2	-2.55	0.000848	down
RN7SL2	-2.62	0	down

Supplementary Table 1 (Continued). Extended table of differentially expressed genes from 2D to 3D. Table of differentially expressed genes upregulated from the 2D to 3D treatment transition. Table lists gene name, log2fc, padjusted value (padj), and direction of dysregulation from 2D to 3D.
