# Supplementary material for: Antibody–Drug Conjugate αEGFR-E-P125A Reduces Triple-negative Breast Cancer Vasculogenic Mimicry, Motility, and Metastasis through Inhibition of EGFR, Integrin, and FAK/STAT3 Signaling
Source: Cancer Res Commun. 2024 Mar 11;4(3):738–56. doi: 10.1158/2767-9764.CRC-23-0278 (PMC10926898; doi:10.1158/2767-9764.CRC-23-0278)
Supplement: Supplementary Table 2 — Extended table of differentially expressed genes from 3D to αEGFR-E-P125A [file crc-23-0278-s15.pdf]

Gene_name	log2FoldChange	padj	Direction
KRTAP2-3	1.8	1E-10	up
ADAMTS6	1.18	0.000003	up
EDN1	1.05	0	up
ST6GALNAC5	1.08	4.8E-09	up
NGF	1.1	0.0125	up
ANKRD1	1.04	0	up
HHIP	0.912	0.0002	up
IL16	0.892	0.0307	up
AC245041.1	0.894	0.00599	up
KLF15	0.857	0.0457	up
FAM196B	0.884	0	up
AC092139.3	0.827	0.0231	up
SERPINE1	0.776	0.000291	up
NRK	0.788	0.00155	up
FST	0.737	0	up
CYR61	0.728	0	up
KISS1	0.767	0	up
RIMS2	0.807	0	up
RIMS1	0.713	0.00467	up
GADD45B	0.648	0	up
DKK1	0.645	0	up
CTGF	0.713	0.0000181	up
CD274	0.648	3.9E-09	up
DOCK2	0.678	0.0429	up
MARCHF4	0.694	0.0000242	up
IGFBP3	0.652	0.00104	up
ADAMTS1	0.65	0.00000251	up
CPED1	0.616	0.000000838	up
IL7R	0.612	0	up
MAP2K6	-0.628	0.00195	down
MYH15	-0.629	0.000354	down
MAP1A	-0.608	0.0346	down
PURPL	-0.603	0.0217	down
ADIRF-AS1	-0.61	0.00000125	down
TNFRSF14	-0.655	0.0417	down
ELF3	-0.711	0.0487	down
CCDC191	-0.66	0.0141	down
ARHGEF37	-0.667	0.00982	down
RORB	-0.693	0.0487	down
ZMIZ1-AS1	-0.668	0.0338	down
SLC9A3-AS1	-0.672	0.00113	down
SBK3	-0.694	0.0497	down
AL133367.1	-0.649	0.00942	down
MYO15B	-0.64	0.0385	down
FSIP2	-0.729	0.0000479	down
CAPN8	-0.759	0.0299	down
AC005077.4	-0.832	0.0361	down
ZNF467	-0.895	0.00564	down
AL391684.1	-0.903	0.0474	down
COL15A1	-1.03	0.0143	down
SLC6A12	-1.13	0.0118	down
ATP6AP1L	-1.19	0.0242	down

Supplementary Table 2. Extended table of differentially expressed genes from 3D to αEGFR-E-P125A. Table of differentially expressed genes dysregulated from the 3D to αEGFR-E-P125A treatment transition. Table lists gene name, log2fc, padjusted value (padj), and direction of dysregulation from 3D to αEGFR-E-P125A.
